# TMEM166 negatively regulates unfolded protein response to affect hepatocellular carcinoma cell growth and sorafenib resistance

**DOI:** 10.1038/s41419-025-08176-w

**Published:** 2025-11-05

**Authors:** Tao Li, Jinqiu Feng, Dan Xia, Yaxin Lou, Pengli Guo, Shufang Ye, Zongming Zhang, Yingyu Chen

**Affiliations:** 1 Department of Immunology, Peking University School of Basic Medical Sciences, NHC Key Laboratory of Medical Immunology, Peking University, Beijing, China; 2 Department of General Surgery, Beijing Electric Power Hospital, Key Laboratory of Geriatrics (Hepatobiliary Diseases), China General Technology Group, Beijing, China; 3 Department of Pathology, Shandong Medical College, Linyi, Shandong China; 4 Medical and Healthy Analytical Center, Peking University, Beijing, China; 5 Center for Human Disease Genomics, Peking University, Beijing, China

**Keywords:** Cancer metabolism, Tumour-suppressor proteins

## Abstract

Transmembrane protein 166 (TMEM166), an endoplasmic reticulum (ER)-resident membrane protein, exerts anticancer effects by inducing autophagy and apoptosis. Although tissues of various cancers downregulate its expression, the biological function of TMEM166 in hepatocellular carcinoma (HCC) remains unclear. Herein, we report that TMEM166 negatively regulates unfolded protein response (UPR) in HCC. TMEM166 was noted to interact with ACSL3 to maintain ACSL3 stability and facilitate lipid storage. *TMEM166* deletion reduced ACSL3 expression and increased lipid utilisation in the mitochondria through fatty acid β-oxidation (FAO), ultimately boosting ATP production. Moreover, *TMEM166*-knockout (KO) cells demonstrated accelerated protein synthesis via the AMPK–mTOR axis. These effects induced sublethal ER stress and UPR activation in *TMEM166*-KO cells. Furthermore, *TMEM166* KO promoted HCC cell proliferation and sorafenib resistance via UPR activity upregulation. We analysed the clinical significance of TMEM166-regulated UPR in human HCC cells and noted that TMEM166 expression was negatively correlated with the activities of UPR-related transcriptional factors such as ATF4, ATF6 and XBP1s in the cells. This study is the first to elucidate the relationship among TMEM166, ER stress, and HCC and may provide and indicate newer avenues for *TMEM166*-targeted gene therapy strategies for HCC treatment.

## Introduction

In eukaryotic cells, the endoplasmic reticulum (ER) governs the synthesis, folding, and processing of one-third of all proteins. When the capacity of ER is affected by physiological demands and pathological perturbations, numerous proteins remain unfolded or misfolded and then accumulate in this organelle; this state is called ‘ER stress’ [1]. To cope with this, cells evoke unfolded protein response (UPR)—an adaptive mechanism for restoring ER protein homeostasis. Based on their ER-located sensors, UPR typically comprises three branches: IRE1, PERK, and ATF6. In resting cells, BiP (also called GRP78) prevents the activation of IRE1, PERK, and ATF6 by constitutively binding to their luminal domains [2]. Under ER stress, unfolded protein and BiP dissociation trigger the dimerization or oligomerisation and trans-autophosphorylation of IRE1 and PERK, as well as the Golgi apparatus translocation and cleavage of ATF6 [3]. Phosphorylated IRE1 induces XBP1s production through precise endonucleolytic cleavage of *XBP1* mRNA, whereas phosphorylated PERK increases ATF4 expression through eIF2α phosphorylation and translation attenuation. XBP1s, ATF4, and cleaved ATF6 then become transported to the nucleus and transcriptionally regulate their target genes [4]. UPR can also be detrimental, promoting apoptosis when the cells are under chronic, unresolved ER stress [5]. As a double-edged sword in cell fate decisions, UPR is crucial in the occurrence of various human diseases, particularly tumor malignant growth, aggressiveness, microenvironment remodeling, and chemotherapy resistance [6–13]. However, the regulatory mechanism of UPR activity in tumor cells warrants further investigation.

Transmembrane protein 166 (TMEM166)—also known as Eva-1 homolog A or family with sequence similarity 176 member A—is an ER membrane protein involved in autophagy and apoptosis [14, 15]. It is expressed in various normal tissues, including those of the liver, gastric, kidney, lung, and pancreas; however, it is downregulated in the corresponding cancer tissues [14, 16]. *TMEM166* overexpression inhibits multiple human cancer cell growth in vitro and in vivo via autophagy and apoptosis [17–20], whereas miR‑103a‑3p-mediated TMEM166 knockdown promotes HCC cell proliferation and migration [21]. TMEM166-mediated autophagy is also involved in neural development [22], ischemic stroke [23], acute liver injury [24] and cardiac homeostasis maintenance [25]. However, the mechanisms underlying the involvement of TMEM166 in the progression of cancers, particularly HCC, remain unclear. As an ER localized protein, TMEM166 may be involved in UPR regulation to affect tumor progression. In the current study, *TMEM166* deletion was noted to increase UPR activity through augmentation of protein translation via signal transduction from ER to mitochondria. In particular, increased UPR activity in *TMEM166*-deleted HCC cells effectively promoted tumor growth but reduced their sorafenib sensitivity.

## Results

### TMEM166 negatively regulates UPR

**Fig. 1:**
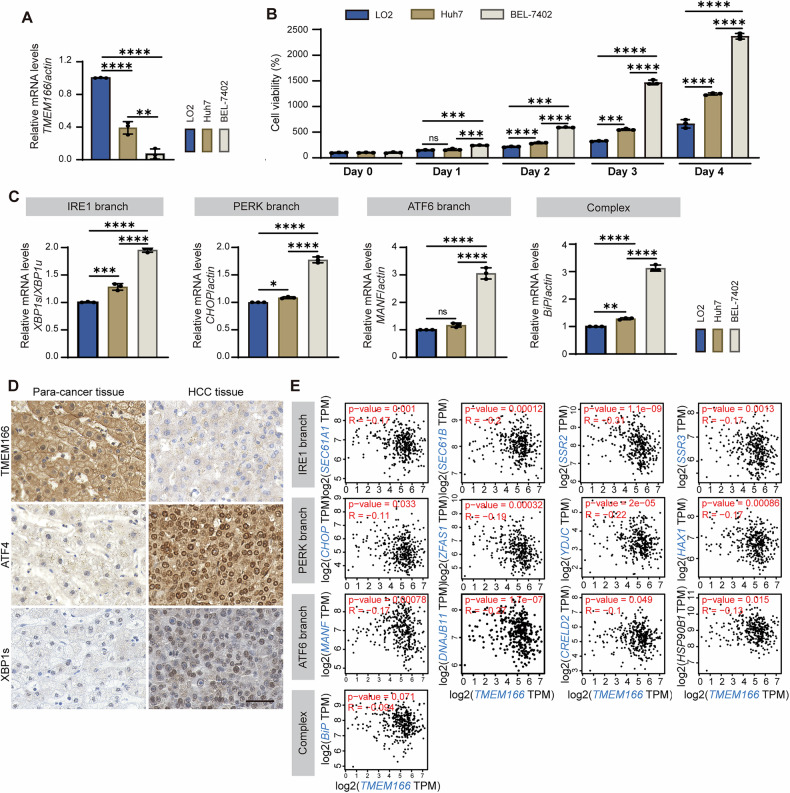
The expression of TMEM166 is negatively related to the UPR activity in HCC. **A** The relative mRNA levels of *TMEM166* in indicated cells were detected by qRT-PCR. **B** The indicated cells were seeded in 96-well plates (1.5 × 10^3^ cells/well; 3 replicates), serum-starved for 24 h and then pulsed with 10% FBS for indicated time. Cell viability was detected by CCK-8 assay. **C** qRT-PCR results of the *XBP1s*/*XBP1u* mRNA ratio, the mRNA levels of *CHOP*, *MANF* and *BiP* in indicated cells. **D** The expression of TMEM166, ATF4 and XBP1s was detected by immunohistochemistry assay. Representative images were shown. Scale bar = 50 µm. **E** Correlation analysis of the levels of *TMEM166* mRNA and UPR targeting genes in HCC. Data are obtained from TCGA and analyzed by using the GEPIA 2. *P < 0.05, **P < 0.01, ***P < 0.001, ****P < 0.0001, ns, no significant. Data (mean ± SD) are representative of at least three independent experiments.

In a study, *TMEM166* was downregulated in HCC tissues and negatively correlated with survival in patients with HCC [20]. Thus, we first assessed the correlation between *TMEM166* expression and cell viability in the hepatocyte line LO2 and hepatoma cell lines Huh7 and BEL-7402 with various differentiation states. As indicated in Fig. 1A, B, cell viability was negatively correlated with *TMEM166* expression. BEL-7402 cells with the lowest *TMEM166* expression were the most viable, followed by Huh 7 cells; in contrast, LO2 cells were the least viable. UPR activation aids cancer cells in adapting to the increased demand for protein and lipid production during rapid proliferation, promoting tumor progression; thus, we subsequently analyzed the relationship between TMEM166 and UPR. Our qRT-PCR results demonstrated that in Huh7 and BEL-7402 cells, the relative mRNA levels of *XBP1s/XBP1u* (IRE1 activation marker), *CHOP* (PERK target gene), *MANF* (ATF6 target gene), and *BiP* (mutual target of all three UPR branches), all higher than those in LO2 cells, were negatively correlated with *TMEM166* expression. As indicated in Fig. 1A, the expression TMEM166 is lowest in BEL-7402, followed by that in Huh7, with the highest expression observed in LO2 cells. In contrast, the UPR activity, indicated by mRNA levels of *XBP1s/XBP1u*, *CHOP*, *MANF* and *BiP*, exhibit an opposite trend (Fig. 1C). To investigate activation of all three UPR branches in human tissues, we initially analyzed the expression for the reported targets of each UPR branch by using the GEPIA 2 database (http://gepia.cancer-pku.cn/) [26]: *SEC61A1*, *SEC61B*, *SSR2*, and *SSR3* for IRE1; *CHOP*, *ZFAS1*, *YDJC*, and *HAX1* for PERK; *MANF*, *DNAJB11*, *CRELD2*, and *HSP90B1* for ATF6; and *BiP* for all three branches. We found that UPR target gene expression was significantly upregulated in HCC tumor tissues compared with normal tissues, indicating that HCC cells demonstrate increased UPR activity (Supplementary Fig. S1). Moreover, our immunohistochemical staining (IHC) results confirmed that *TMEM166* expression was lower in liver cancer tissues than in adjacent noncancerous tissues (Fig. 1D)—consistent with a previous report [20]. We detected strong ATF4 and XBP1s protein signals, mainly localized in the nucleus, in HCC tissue samples but weak or no signal in adjacent noncancerous tissue samples (Fig. 1D). Furthermore, by analyzing the expression of UPR target genes, we found that *TMEM166* expression was negatively correlated with UPR activity in HCC tumor tissues (Fig. 1E). Thus, *TMEM166* expression might be associated with UPR activity in HCC cells.

**Fig. 2:**
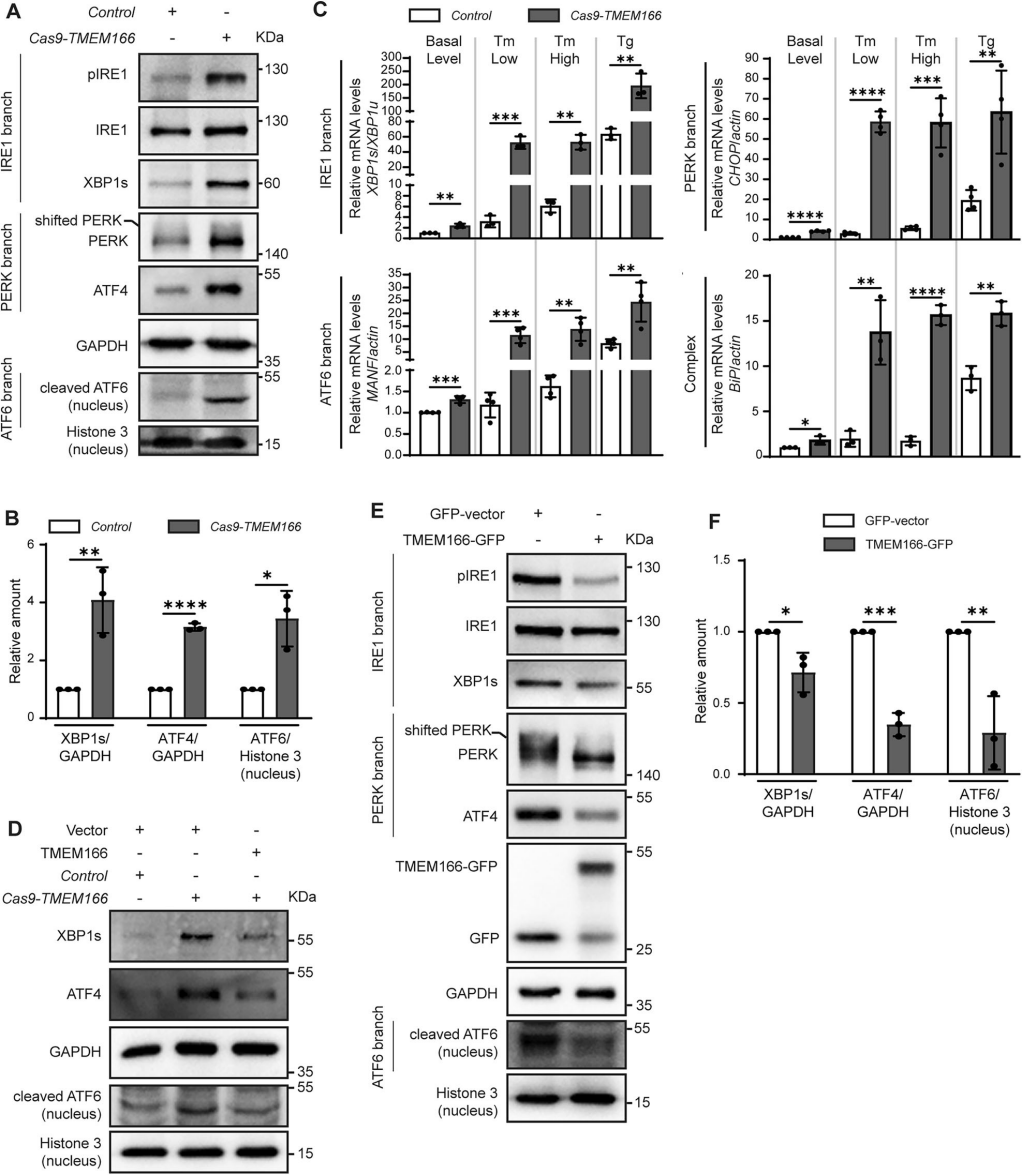
TMEM166 negatively regulates UPR. **A** Immunoblotting of the indicated UPR proteins in whole cell lysates or nuclear lysates extracted from *Control* and Cas9-*TMEM166*/Huh7 cells. Phosphorylation of PERK is recognized as a band shift to a higher molecular weight. **B** Quantification of XBP1s, ATF4 and nuclear cleaved ATF6 relative to GAPDH or nuclear Histone 3 in *Control* and Cas9-*TMEM166*/Huh7 cells. Average value in *Control* cells was normalized as 1. **C** *Control* and Cas9-*TMEM166*/Huh7 cells treated with or without Tm (0.5 μg/mL, 6 h, 1 μg/mL, 6 h) or Tg (0.2 μM, 6 h). The relative mRNA levels of the indicated genes were detected by qRT-PCR. **D** Immunoblotting of the indicated UPR proteins in *Control* and Cas9-*TMEM166*/Huh7 cells stably expressing vector or TMEM166. **E** BEL-7402 cells were transfected with indicated plasmids for 48 h, then the indicated UPR proteins in whole cell lysates or nuclear lysates were detected by immunoblotting. **F** Quantification of XBP1s, ATF4 and nuclear ATF6 relative to GAPDH or nuclear Histone 3 in indicated cells. Average value in BEL-7402 cells over-expressing GFP-vector was normalized as 1. *P < 0.05, **P < 0.01, ***P < 0.001, ****P < 0.0001. Data (mean ± SD) are representative of at least three independent experiments.

To explore the effects of TMEM166 on UPR activity in HCC cells further, we generated *TMEM166-*KO Huh7 cells through *Cas9-CRISPR* genome editing (Supplementary Fig. S2A). We confirmed that *TMEM166* was knocked out in Cas9-*TMEM166* Huh7 cells through genotyping and qRT-PCR (Supplementary Fig. S2A, S2B). Next, we measured the activities of all three UPR branches with or without ER stress inducers. We noted that *TMEM166* KO upregulated UPR molecules, including phosphorylated IRE1(S724), XBP1s, phosphorylated PERK (recognized as a band shift to higher molecular weight), ATF4, and nuclear-located cleaved ATF6 in resting Huh7 cells (Fig. 2A, B). Simultaneously, *XBP1s/XBP1u*, *CHOP, MANF*, and *BiP* mRNA expression was higher in *TMEM166*-KO Huh7 cells than in wildtype (WT) cells (Fig. 2C), indicating an increase in UPR activity. Rescue of *TMEM166* expression resulted in the inhibition of UPR activation in *TMEM166*-KO Huh7 cells (Fig. 2D). In BEL-7402 cells, *TMEM166* overexpression led to downregulation in UPR activity (Fig. 2E, F).

Tunicamycin (Tm) and thapsigargin (Tg) are commonly used to induce cellular ER stress. Regardless of the presence of Tm, *TMEM166*-KO Huh7 cells significantly increased *XBP1s/XBP1u*, *CHOP*, *MANF*, and *BiP* mRNA expression (Fig. 2C). Similar results were obtained in Tg-treated Cas9-*TMEM166* Huh7 cells (Fig. 2C). Therefore, *TMEM166* expression might result in negative modulation of UPR activity and viability in hepatoma cells.

### *TMEM166* deletion accelerates protein synthesis to induce ER stress

**Fig. 3:**
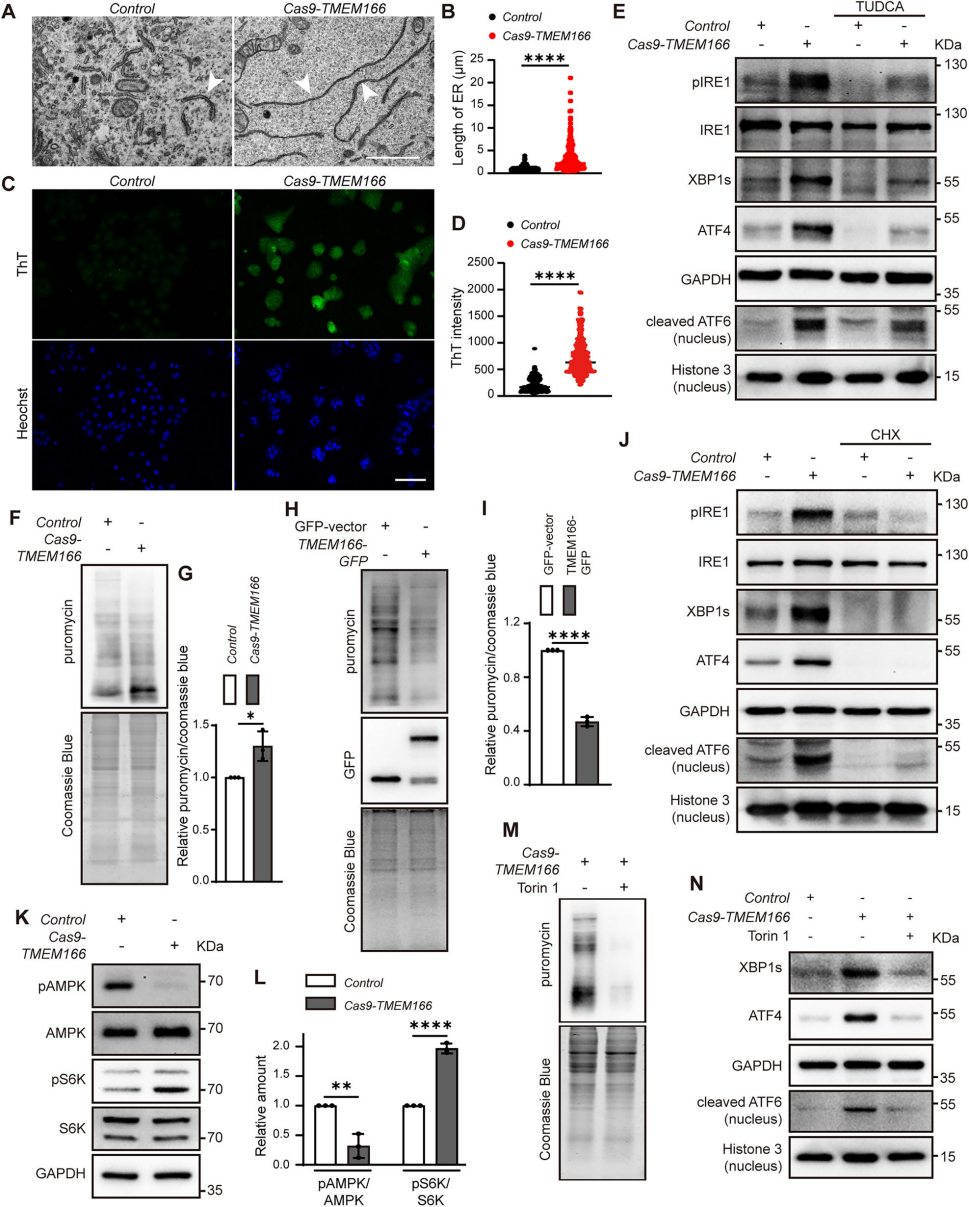
*TMEM166* deletion induces ER stress to activate UPR via AMPK-mTOR axis. **A** Representative TEM images in *Control* and Cas9-*TMEM166*/Huh7 cells. Arrowheads indicate the ER. Scale bars, 2 μm. **B** Quantification of ER length in indicated cells. Data are means ± SD of results from 183 ER in 12 *Control* cells and 185 ER in 10 Cas9-*TMEM166* cells, respectively. **C** *Control* and Cas9-*TMEM166*/Huh7 cells were stained with ThT (20 μM). Representative fluorescence images were showed. Scale bars, 100 μm. **D** Quantification of ThT fluorescence intensity. Data are means ± SD of results from 640 *Control* or Cas9-*TMEM166* cells. **E** The different cells were treated with TUDCA (200 μM, 30 h). The indicated UPR proteins were detected by immunoblotting. **F**, **H** Assessment of protein synthesis in indicated cells by SUnSET assay. **G**, **I** Quantification of puromycin incorporation relative to Coomassie blue staining in cells. Average value in *Control* cells or over-expressing GFP-vector cells was normalized as 1. Data (mean ± SD) are representative of at least three independent experiments. **J** *Control* and Cas9-*TMEM166*/Huh7 cells were treated with CHX (10 μg/mL, 24 h). The indicated UPR proteins were detected by immunoblotting. **K** Immunoblotting of the indicated proteins in *Control* and Cas9-*TMEM166*/Huh7 cells. **L** Quantification of phosphorylation of AMPK and S6K relative to total protein in *Control* and Cas9-*TMEM166*/Huh7 cells. Average value in *Control* cells was normalized as 1. Data (mean ± SD) are representative of at least three independent experiments. **M** Cas9-*TMEM166*/Huh7 cells were treated with or without Torin (0.5 μM, 24 h). The protein synthesis was assessed by SUnSET assay. **N** Cas9-*TMEM166*/Huh7 cells were treated with or without Torin (0.5 μM, 24 h). The indicated UPR proteins were detected by immunoblotting. *P < 0.05, **P < 0.01, ****P < 0.0001.

UPR can be activated in an ER stress-dependent or -independent manner [2]. The colocolization of TMEM166 and PDI indicated that TMEM166 is an ER-resident protein (Fig. S4A). We hypothesized that *TMEM166* deletion perturbs ER homeostasis, activating UPR. Chronic disruption of ER proteostasis changes ER morphology; thus, we first examined the ultramicrostructure of ER in WT and *TMEM166*-KO Huh7 cells through transmission electron microscopy. As shown in Fig. 3A, B, ER was more elongated in the *TMEM166*-KO cells than in the WT cells, indicating that *TMEM166* deletion alters the ER structure. Thioflavin T (ThT) is a small molecule, that exhibits enhanced fluorescence when it binds to protein aggregates [27]. Fluorescence microscopy demonstrated that significantly enhanced ThT signals were observed in *TMEM166*-KO cells compared with the WT cells (Fig. 3C, D). Moreover, treatment with the ER stress inhibitor tauroursodeoxycholic acid (TUDCA) significantly reduced UPR activity in *TMEM166* KO cells. This reduction was evidenced by decreased IRE1 phosphorylation, lower levels of XBP1s and ATF4 production, and diminished nuclear localization of cleaved ATF6 in *TMEM166* KO cells treated with TUDCA (Fig. 3E). The above results suggest that *TMEM166* deletion may increase protein aggregation accompanied by ER stress.

To explore the underlying mechanism further, we analyzed the transcriptome in the WT and *TMEM166*-KO cells. *TMEM166* deletion was noted to upregulate multiple genes related to protein synthesis (Supplementary Fig. S3A, S3B). By using the SuNSET assay [28] and RNA-seq, *TMEM166* deletion was noted to enhance protein translation, but TMEM166 overexpression was observed to inhibit it (Fig. 3F–3I). Moreover, the protein translation inhibitor CHX inhibited UPR activation in the *TMEM166*-KO cells (Fig. 3J).

The mTOR/S6K cascade positively regulates mRNA translation and ribosome biosynthesis, and AMPK suppresses mTORC1 activity [29, 30]. The *TMEM166*-KO cells demonstrated upregulation of S6K phosphorylation but had significantly downregulated AMPK phosphorylation (Fig. 3K, L). In addition, mTOR activity inhibition by torin (a potent mTOR inhibitor) reduced protein translation and UPR activity in the *TMEM166*-KO cells (Fig. 3M, N). Taken together, these results indicated that *TMEM166* deletion might increase protein translation to induce ER stress, which may be associated with the AMPK–mTOR axis.

### TMEM166 deletion promotes mitochondrial ATP production

**Fig. 4:**
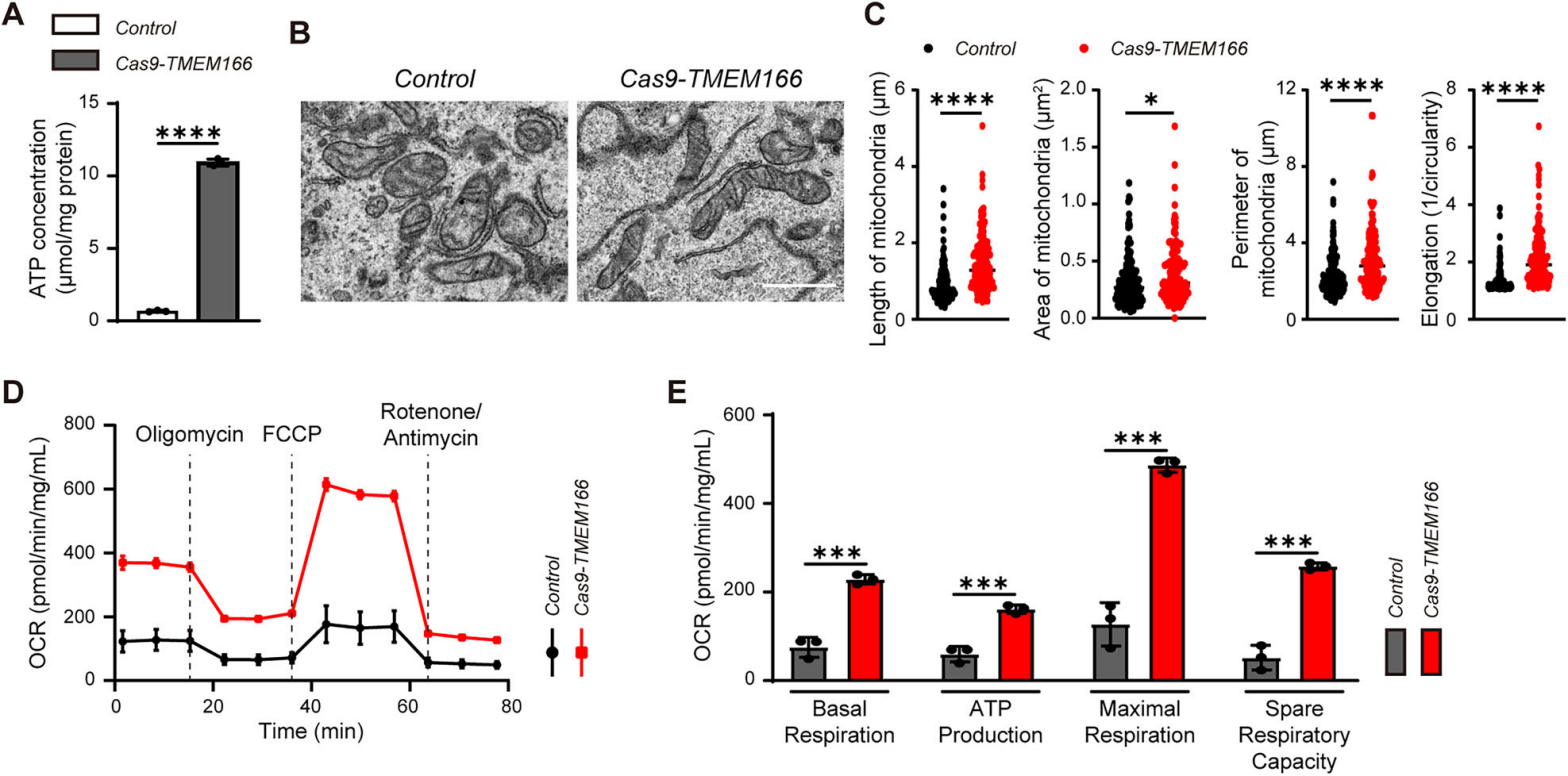
*TMEM166* deletion stimulates mitochondrial ATP production. **A** Detection of ATP concentration in *Control* and Cas9-*TMEM166*/Huh7 cells. **B** Representative TEM images in *Control* and Cas9-*TMEM166*/Huh7 cells. Scale bars, 1 μm. **C** Quantification of mitochondria length obtained from 126 mitochondria in 11 *Control* cells and 140 mitochondria in 9 Cas9-*TMEM166* cells; quantification of mitochondria area and perimeter from 161 mitochondria in 11 *Control* cells and 167 mitochondria in 9 Cas9-*TMEM166* cells; elongation factor from 161 mitochondria in 11 *Control* cells and 166 mitochondria in 9 *Cas9-TMEM166* cells. **D** Plots of the Oxygen consumption rate (OCR) normalized to the amount of protein in *Control* and Cas9-*TMEM166*/Huh7 cells. **E** Basal respiration, ATP production, proton leak, and maximal respiration in *Control* and Cas9-*TMEM166*/Huh7 cells. *P < 0.05, ***P < 0.001, ****P < 0.0001. Data (mean ± SD) are representative of at least three independent experiments.

As a central energy sensor, AMPK becomes activated in response to low ATP/AMP levels [31]. Consistently, the *TMEM166*-KO cells demonstrated high cellular ATP levels (Fig. 4A). Transmission electron microscopy revealed that the *TMEM166*-KO cells had elongated mitochondria with undisrupted cristae (Fig. 4B, C). Thus, we hypothesized that TMEM166 promotes mitochondrial respiration, increasing cellular ATP levels. Based on their mitochondrial oxygen consumption rates, the *TMEM166*-KO cells demonstrated elevated mitochondrial respiration and ATP production (Fig. 4D, E). Therefore, *TMEM166* deletion might promote mitochondrial ATP production, in turn inhibiting AMPK activation and downstream events.

### TMEM166 interacts with ACSL3, maintains its stability and decelerates FAO

**Fig. 5:**
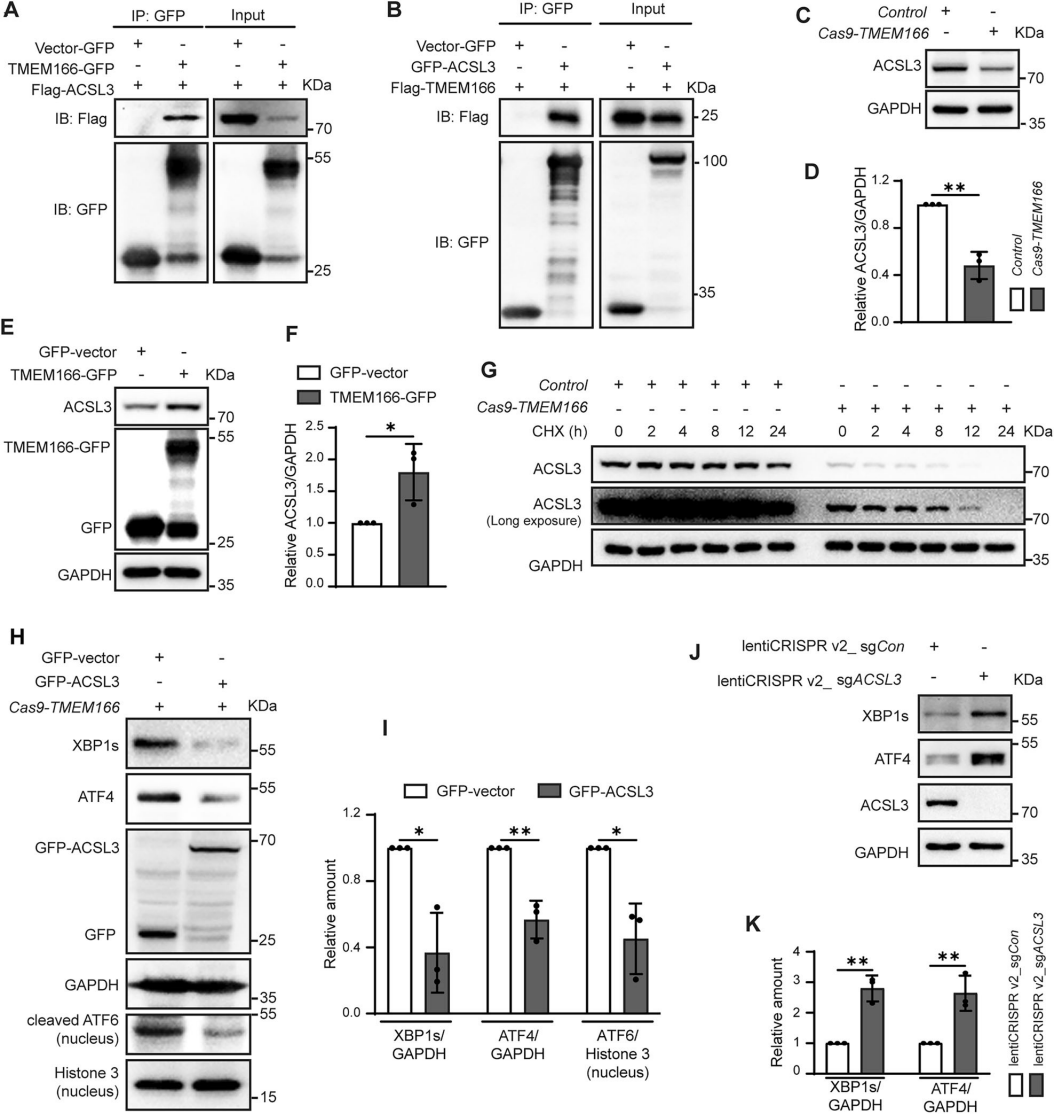
TMEM166 interacts with ACSL3 and positively regulates its hemostasis. **A**, **B** HEK293T cells were transfected with indicated plasmids for 24 h, then cell lysates were subjected to IP using an anti-GFP beads. Flag-ACSL3 (**A**) or Flag-TMEM166 (**B**) were detected in the immunoprecipitates by western blotting. **C** The levels of ACSL3 in the indicated cells were detected by Immunoblotting. **D** Quantification of ACSL3 relative to GAPDH in *Control* and Cas9-*TMEM166*/Huh7 cells. Average value in *Control* cells was normalized as 1. **E** Huh7 was transfected with indicated plasmids for 24 h, the levels of ACSL3 were detected by immunoblotting. **F** Quantification of ACSL3 relative to GAPDH. Average value in GFP-vector-transfected cells was normalized as 1. **G** The indicated cells were incubated with cycloheximide (CHX, 250 μg/mL) for different time. The levels of ACSL3 were detected by Immunoblotting. **H** *TMEM166* KO Huh7 was transfected with indicated plasmids for 24 h, the levels of indicated protein in whole cell lysates or nuclear lysates were detected by immunoblotting. **I** Quantification of XBP1s, ATF4 and nuclear cleaved ATF6 relative to GAPDH or nuclear Histone 3. Average value in GFP-vector-transfected cells was normalized as 1. **J** The levels of XBP1s and ATF4 were detected by immunoblotting in the indicated Huh7 cells. **K** Quantification of the indicated protein relative to GAPDH. Average value in the stable lentiCRISPR v2-sg*Con*-expressing Huh7 cells was normalized as 1. *P < 0.05, **P < 0.01. Data (mean ± SD) are representative of at least three independent experiments.

The results of immunofluorescence staining revealed that TMEM166 is an ER-resident protein instead of mitochondrial protein, since it could colocalized with PDI, an ER marker, instead of Mito-tracker (Fig. S4A, B). We next assessed the molecular mechanisms underlying the effects of TMEM166 on mitochondrial respiration. We first explored the potential interactions of TMEM166 through immunoprecipitation (IP)-mass spectrometry. Among all TMEM166-binding proteins, we next focused on ACSL3, an ER-resident isozyme of the long-chain fatty-acid-coenzyme A ligase family [32]. Data from a co-immunoprecipitation (co-IP) assay demonstrated that association of TMEM166 with ACSL3 was detected in HEK293T cells by reciprocal immunoprecipitations (Fig. 5A, B); simultaneously, immunofluorescence staining revealed that TMEM166 was colocalised with ACSL3 (Supplementary Fig. S4C). Thus, both TMEM166 and ACSL3 were present as a complex in the cells.

We further investigated the biological significance of TMEM166–ACSL3 interactions and found that *TMEM166* KO decreased ACSL3 protein levels (Fig. 5C, D), whereas *TMEM166* overexpression increased it (Fig. 5E, F). We subsequently analyzed the half-life of ACSL3 protein by using CHX. As illustrated in Fig. 5G, *TMEM166* KO promoted endogenous ACSL3 decay, suggesting that TTMEM166 is a positive regulator of ACSL3 stabilization. Moreover, we assessed the association between ACSL3 and UPR activity. ACSL3 overexpression inhibited UPR activation in *TMEM166* KO/Huh7 cells (Fig. 5H, I), while similar to *TMEM166* KO, *ACSL3* deletion increased UPR activity under basal conditions in wild-type Huh7 cells, as evidenced by increased XBP1s and ATF4 expression (Fig. 5J, K).

**Fig. 6:**
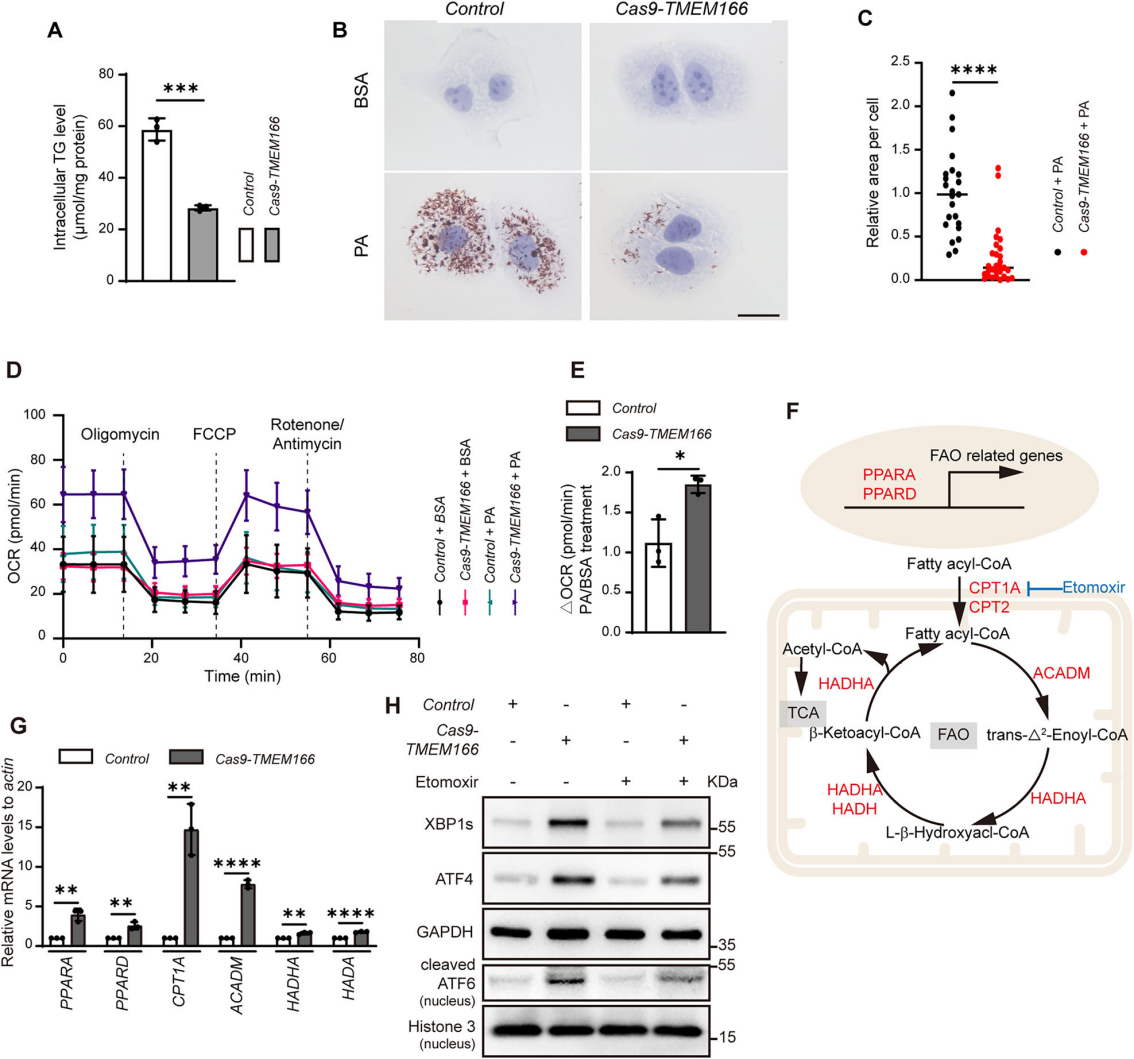
*TMEM166* deletion decreases lipid storage and promotes FAO. **A** The levels of triglyceride (TG) in *Control* and Cas9-*TMEM166*/Huh7 cells. **B** *Control* and Cas9-*TMEM166*/Huh7 cells were treated with or without PA (0.2 mM, 6 h), and stain with Oil Red O (ORO). Representative images of ORO staining. Scale bars, 20 μm. **C** Quantification of relative area of lipid droplets in *Control* and Cas9-*TMEM166*/Huh7 cells. Average value in *Control* cells was normalized as 1. Data are means ± SD of results from 23 images of *Control* cells and 29 images of Cas9-*TMEM166* cells. **D** Detection of OCR in *Control* and Cas9-*TMEM166*/Huh7 cells treated with BSA or PA in substrate-limited medium. **E** Quantification of △OCR (PA-treated OCR/BSA-treated OCR). Data are means ± SD of results from 3 experiments. **F** Schematic depiction of the regulation of FAO-related genes and the FAO process. **G** The mRNA levels of FAO-linked genes in *Control* and Cas9-*TMEM166*/Huh7 cells were detected by qRT-PCR. Data are means ± SD of results from 3 experiments. **H** *Control* and Cas9-*TMEM166*/Huh7 cells were treated with Etomoxir (20 μM, 24 h). The indicated UPR proteins were detected by immunoblotting. *P < 0.05, **P < 0. 01, ****P < 0.0001.

ACSL3 mainly facilitates lipid storage in lipid droplets (LDs) budding from ER, and downregulation of ACSL3 activity stimulates FAO [32, 33]. FAO and mitochondrial oxidative phosphorylation (OXPHOS) are interdependent processes that maintain the cellular energy balance in combination. Through triglyceride (TG) breakdown, FAO provides essential electron carriers (NADH and FADH2) and substrates (acetyl-CoA) for OXPHOS, whereas OXPHOS uses these electron carriers and substrates to produce ATP, the cellular energy currency [34]. Similarly, *TMEM166* deletion reduced endogenous TG content (Fig. 6A). Palmitate (PA), an FAO substrate, induced fewer LDs in the *TMEM166*-KO cells than in the WT cells (Fig. 6B, C). Next, we measured the oxygen consumption rate after cell treatment with bovine serum albumin (BSA) or PA in a substrate-limited medium and found that ΔOCR between BSA- and PA-treated cells was higher in the *TMEM166*-KO cells than in the WT cells (Fig. 6D, E), suggesting that *TMEM166* deletion may increase FAO rates. Moreover, both RNA-seq (Supplementary Fig. S3C) and qRT-PCR (Fig. 6F, G) revealed that multiple FAO-related genes were upregulated in the *TMEM166*-KO cells. Furthermore, FAO inhibition mediated by etomoxir (a CPT1A inhibitor) decreased UPR activity in the *TMEM166*-KO cells (Fig. 6H). Taken together, our data demonstrated that TMEM166 positively regulates ACSL3 stabilization, and that decreased ACSL3 expression in the *TMEM166*-KO cells promoted FAO and then mitochondrial ATP production, initiating UPR.

### *TMEM166* KO promotes proliferation and sorafenib resistance in Huh7 cells via UPR

**Fig. 7:**
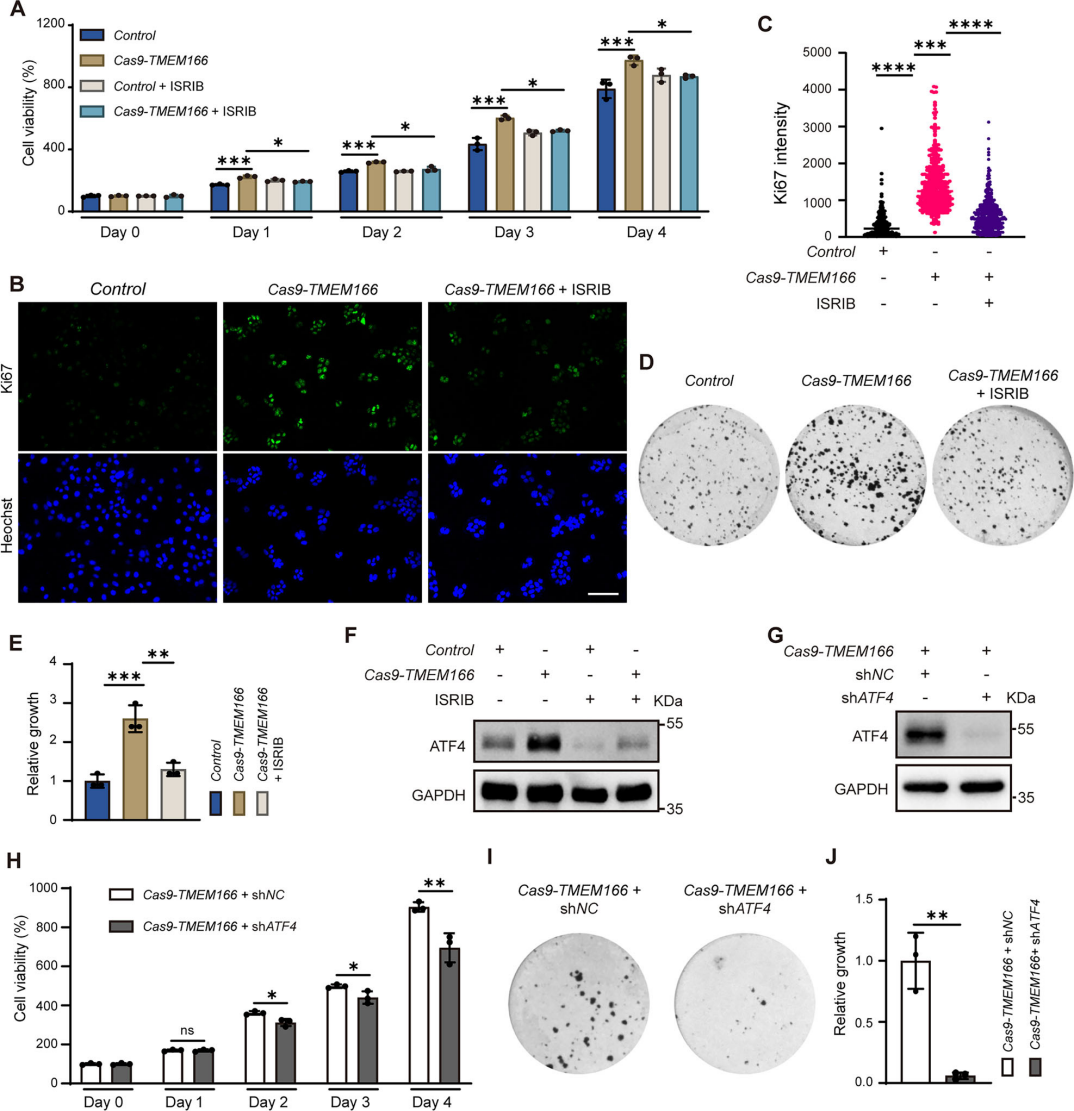
*TMEM166* deletion promotes Huh7 cell proliferation by upregulating ATF4. **A** *Control* and Cas9-*TMEM166*/Huh7 cells were serum starved for 24 h and then seeded in 96-well plates (1.5 × 10^3^ cells/well; 3 replicates), pulsed with 10% FBS and treated with or without ISRIB (1 μM) for indicated time. Cell viability was detected by CCK-8 assay. **B** The indicated cells were treated as (**A**), then stained with anti-Ki67 antibody. Representative fluorescence images were shown. Scale bars, 100 μm. **C** Quantification of Ki67 fluorescence intensity. Data are means ± SD of results from 400 cells. **D** Clonal formation of *Control* and Cas9-*TMEM166*/Huh7 cells treated with or without ISRIB (1 μM). Representative images of colony formation by indicated cells. **E** Relative area of clones. Average value in *Control* cells was normalized as 1. **F** *Control* and Cas9-*TMEM166*/Huh7 cells were treated with ISRIB (1 μM, 24 h). The level of ATF4 was detected by immunoblotting. **G** The level of ATF4 in the indicated cells were measured by Immunoblotting. **H** The indicated cells were serum starved for 24 h, then seeded in 96-well plates (1.5 × 10^3^ cells/well; 3 replicates), pulsed with 10% FBS for indicated time. Cell viability was detected by CCK-8 assay. **I** Clonal formation of the indicated cells. Representative images of colony formation by indicated cells. **J** Relative area of clones. Average value in the *shNC*-expressing Cas9-*TMEM166*/Huh7 cells was normalized as 1. *P < 0.05, **P < 0.01, ***P < 0.001, ****P < 0.0001, ns, no significant. Data (mean ± SD) are representative of at least three independent experiments.

We subsequently analyzed the relationship among TMEM166, UPR, and HCC cell proliferation. Our CCK8 assay revealed that *TMEM166* overexpression reduced viability in BEL-7402 cells (Supplementary Fig. S5A). However, *TMEM166* KO significantly promoted Huh7 cell proliferation, as indicated by increased cell viability (Fig. 7A), Ki67 fluorescence intensity (Fig. 7B, C), and clonal formation (Fig. 7D, E). TUDCA treatment reduced viability in the *TMEM166*-KO cells (Supplementary Fig. S5B), indicating that increased UPR activity might promote *TMEM166*-KO cell proliferation.

Cancer cells can exploit UPR to promote proliferation and metastasis, mainly via the downstream transcriptional factors XBP1s, ATF4, and cleaved ATF6 [7, 11, 35–38]. We then assessed the contribution of each UPR branch and its downstream transcriptional factor to the proliferative effect in the *TMEM166*-KO cells via pharmacological or genetic manipulation. We noted that ISRIB inhibited ATF4 expression (Fig. 7F). Both ISRIB and *ATF4* knockdown (Fig. 7G) significantly reduced cell viability, Ki67 fluorescence intensity, and clonal formation in the *TMEM166*-KO cells (Fig. 7A–E, H–J). However, knockdown of *XBP1* and *ATF6* did not affect clonal formation in the *TMEM166*-KO cells (Supplementary Fig. S5C–S5F). Thus, among all the three UPR branches, the PEKR-ATF4 axis is key in *TMEM166*-KO cell proliferation.

**Fig. 8:**
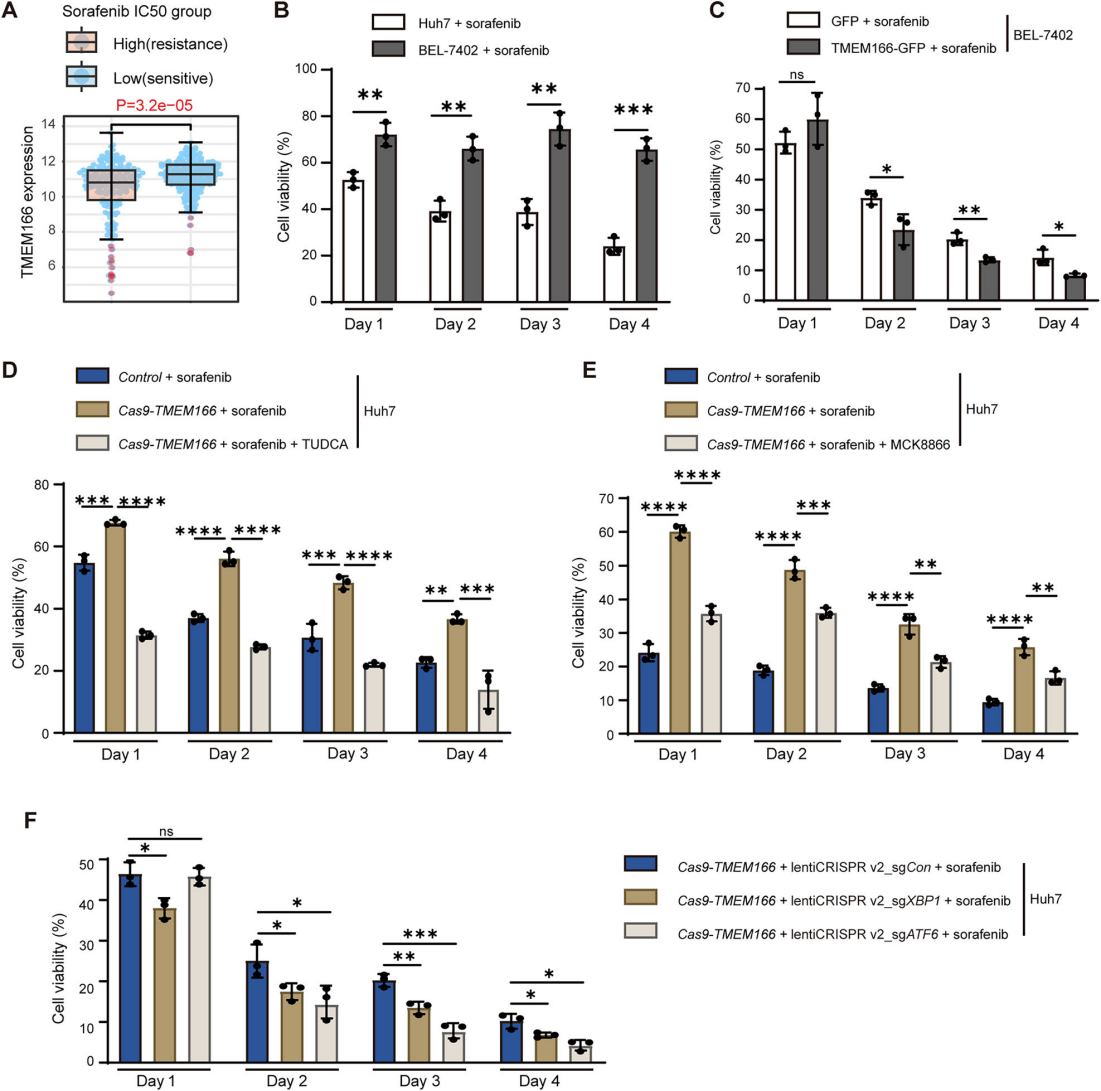
*TMEM166* deficiency increases sorafenib resistant by activating IRE1 and ATF6 branch in Huh7 cells. **A** *TMEM166* expression in sorafenib-resistant or -sensitive HCC group. Data are obtained from TCGA and analyzed by using the CADSP. **B** Huh7 or BEL-7402 cells were seeded in 96-well plates (3 × 10^3^ cells/well; 3 replicates) and treated with sorafenib (5 μM) for indicated time. Cell viability was detected by CCK-8 assay. **C** BEL-7402 cells transfected with vector-GFP or TMEM166-GFP were seeded in 96-well plates (1.5 × 10^3^ cells/well; 3 replicates), then treated with sorafenib (5 μM) for indicated time. Cell viability was detected by CCK-8 assay. **D** Different cells were seeded in 96-well plates (3 × 10^3^ cells/well; 3 replicates), treated with sorafenib (5 μM) and with or without TUDCA (200 μM) for indicated time. Cell viability was detected by CCK-8 assay. **E** Different cells were seeded in 96-well plates (3 × 10^3^ cells/well; 3 replicates), incubated with sorafenib (5 μM) and with or without MCK8866 (1 μM) for indicated time. Cell viability was detected by CCK-8 assay. **F** The indicated cells were seeded in 96-well plates (3 × 10^3^ cells/well; 3 replicates), treated with sorafenib (5 μM) for indicated time. Cell viability was detected by CCK-8 assay. *P < 0.05, **P < 0.01, ***P < 0.001, ****P < 0.0001, ns, no significant. Data (mean ± SD) are representative of at least three independent experiments.

HCC patients treated with sorafenib, a first-line standard therapeutic for advanced HCC [39], frequently acquire resistance [40]. Because the mechanism underlying sorafenib resistance remains unknown, we explored the effects of TMEM166 on sorafenib sensitivity via UPR downregulation in patients with HCC. The data from the Comprehensive Pancancer Analysis of Drug Sensitivity database (https://smuonco.shinyapps.io/CPADS/ or https://robinl-lab.com/CPADS) indicated that low *TMEM166* expression is correlated with increased sorafenib resistance (Fig. 8A). Similarly, we found that sorafenib-treated *TMEM166* low-expressing BEL-7402 cells were more sorafenib resistant than *TMEM166* high-expressing Huh7 cells (Fig. 8B). Nevertheless, *TMEM166* overexpression sensitized BEL-7402 cells to sorafenib treatment (Fig. 8C), whereas *TMEM166* deletion induced sorafenib resistance in Huh7 cells (Fig. 8D). Furthermore, alleviating ER stress through TUDCA treatment sensitized *TMEM166*-KO cells to sorafenib (Fig. 8D), indicating that UPR activation contributes to sorafenib resistance.

We subsequently analyzed the contribution of each UPR branch in the *TMEM166*-KO cells. MCK8866-mediated inhibition of IRE1 RNase activity (Fig. 8E) and knockdown of *XBP1* (Fig. 8F) both sensitized the *TMEM166*-KO cells to sorafenib. Similar results were obtained in *ATF6* knockdown cells (Fig. 8F). Notably, neither ISRIB nor ATF4 knockdown affected the sorafenib sensitivity of the *TMEM166*-KO cells (Supplementary Fig. S6A, S6B). Taken together, the results indicated that *TMEM166* deletion promotes cell proliferation dependent on PERK activation and that IRE1 and ATF6 activation confers HCC cells with sorafenib resistance.

## Discussion

In the present study, we identified TMEM166 as a negative regulator of UPR for the first time. Mechanistically, TMEM166 interacts with and stabilizes ACSL3 to maintain lipid storage. In the absence of TMEM166, ACSL3 downregulation accelerates FAO, promoting mitochondrial ATP production and activating mTOR via dephosphorylation of AMPK. Subsequently, mTOR activation promotes protein synthesis, inducing sublethal ER stress and initiating UPR. Functionally, in hepatoma cells, *TMEM166* deletion promotes cell proliferation via the PERK–ATF4 axis and confers sorafenib resistance via IRE1 and ATF6 activation.

We previously reported that TMEM166, a lysosome and an ER-associated membrane protein, interacts with ATG16L1 to induce autophagosome formation [14, 15]. TMEM166, via both autophagic and apoptotic mechanisms, plays a crucial role in numerous physiological and pathological processes, including embryonic neurogenesis, rapid heart failure development, acute liver injury, and HBV replication [22, 24, 25, 41]. In cancer progression, TMEM166 inhibits growth and induces death in the cells of non–small-cell lung cancer, glioblastoma, and HCC [18–20]. In HCC, TMEM166 overexpression inhibits cell proliferation, migration, and invasion via TP53 upregulation [20]. In the current study, we overexpressed *TMEM166* in low-expressing HCC cells and deleted it in high-expressing HCC cells and found that TMEM166 negatively regulate UPR to inhibit HCC progression and increase sorafenib sensitivity. So, TMEM166 may influence the growth of HCC cells through a variety of mechanisms. Our results supplement the current knowledge on the biological function of TMEM166 and refine the mechanism through which TMEM166 regulates liver cancer development.

As the organelles highly interconnected with ER, mitochondria play a vital role in UPR activity. ER stress–induced cell death mainly depends on the core mitochondrial apoptosis pathway [4]; moreover, mitochondria are involved in UPR regulation. For instance, mitochondrial dysfunction can inhibit UPR activation through attenuation of global protein translation [42]. PIGBOS, a mitochondrial outer membrane–located protein, modulates UPR at ER–mitochondria contact sites [43]. In the present study, we found that *TMEM166* deletion activates UPR through signal transduction between ER and mitochondria. In particular, TMEM166 interacts with and stabilizes ACSL3 in ER. ACSL3 has mainly been implicated in lipid anabolism via fatty acid activation. In *TMEM166*-KO cells, lipid storage inhibition might facilitate lipid transport to mitochondria and stimulate FAO [33]. Here, increased FAO activity promoted mitochondrial respiration and ATP supply, increasing protein synthesis burden in ER and inducing sublethal ER stress to activate UPR via the AMPK–mTOR axis. As such, our results for the first time indicated that *ACSL3* knockdown phenocopies the effects of *TMEM166* deletion on UPR activity and that FAO and mTOR pathway inhibition inhibits UPR activation in *TMEM166*-KO cells. Taken together, our results further expand the study of mitochondrial role in UPR modulation and link lipid metabolism with UPR regulation.

Cancer cells, including HCC cells, are often exposed to ER stress because of extrinsic and intrinsic perturbations, such as hypoxia, low pH, nutrient deprivation, sustained secretory pathway demands, and somatic mutations in target proteins [6, 12, 44]. Although irreversible ER stress is detrimental to cancer cells, pharmacological and remediable ER stress can promote cellular adaption to a harsh environment and drug resistance through UPR initiation [10, 45]. For instance, during cancer progression, the IRE1–XBP1s axis intrinsic to melanoma cells drives immunosuppression by promoting cholesterol production and reprogramming myeloid-derived suppressor cells [10]. ATF4, the downstream transcriptional factor of PERK, promotes fructolysis under glucose-deprived conditions in glioblastoma, supporting cell proliferation [8].

Sorafenib treatment is considered a main strategy for advanced HCC. However, more than half of the patients do not benefit from it in the initial treatment phases, and most patients with effective treatment outcomes initially acquire drug resistance within 6 months through tumor microenvironment and tumor cell–intrinsic epigenetics, transport processes, and regulated cell death rewiring [46]. Emerging evidence also indicates that sustained UPR activation confers sorafenib resistance to cancer cells [40, 47–49]. For instance, the IRE1-XBP1s axis is crucial for sorafenib resistance via c-MYC signaling activation [48]. By performing an IHC assay in human tissues and analyzing publicly available human HCC datasets, we confirmed that UPR activity is elevated in HCC cells and negatively correlated with *TMEM166* expression. Moreover, *TMEM166* expression was lower in sorafenib-resistant HCC tissue samples than in those sensitive to sorafenib. According to the current and previous results, TMEM166 has antiproliferative effects in tissues of various cancers, including HCC [17, 20]. Moreover, our results for the first time indicated that modulation of *TMEM166* expression can affect sorafenib resistance in HCC cells. We also noted that alleviating ER stress with TUDCA inhibited tumor cell proliferation and elevated sorafenib sensitivity in *TMEM166*-KO hepatoma cells, demonstrating that UPR activation is essential for them. Notably, by deciphering the contribution of all three UPR branches and their downstream transcriptional factor, we observed that different branches played distinct roles in the proliferation and sorafenib resistance in *TMEM166*-KO cells. The PERK–ATF4 axis is key in *TMEM166*-KO cell proliferation, consistent with the strong ATF4 immunoreactivity in HCC tissue samples. Moreover, activation of IRE1 and ATF6 but not that of PERK reduced sorafenib sensitivity in *TMEM166*-KO cells. Recently, Liu *et al* reported that TMEM166 is down-regulated in Lenvatinib-resistant HCC cells, and *TMEM166* knockdown induces lenvatinib resistance in HCC cells while TMEM166 overexpression reverses it in vitro and in vivo [50]. Despite their distinct mechanisms of action, Lenvatinib and sorafenib are both tyrosine kinase inhibitors which are used as first-line treatments for HCC. These findings indicated that in addition to being a prognostic marker for HCC progression, TMEM166 maybe a biomarker of chemotherapy-resistance. Moreover, combined with sorafenib treatment, *TMEM166* expression manipulation via gene therapy might be a novel treatment strategy for advanced HCC.

### Limitations of this study

TMEM166 is down-regulated in liver cancer cells lines and biopsy samples from HCC patients. By modulation the expression of TMEM166, we defined that TMEM166 could negatively regulated UPR activity to affect cell growth and the sensitivity to sorafenib treatment in liver cancer cells. Developing preclinical mouse HCC models are likely to provide a more comprehensive analysis of TMEM166 expression and its relationship with UPR in HCC progression. And the underlying mechanisms by which oncogene stress is integrated into TMEM166 expression also need to be further elucidated.

## Materials and methods

### Antibodies and reagents

The antibodies and major reagents used in this study are listed in Supplementary Table 1.

### Plasmid construction

The plasmid containing the *TMEM166* sgRNA for CRISPR interference were designed and constructed by Shanghai Biomodel Organism Science & Technology Development Co., Ltd (China). *lentiCRISPR v2* was a gift from Feng Zhang (Addgene plasmid # 52961; http://n2t.net/addgene:52961; RRID:Addgene_52961). *pLKO.1_shNC* and *pLKO.1*_*shATF4* plasmid were the kind gifts from Dr. Likun Wang (Institute of Biophysics, CAS, China). And the sgRNA and shRNA sequences were listed in Supplementary Table 2.

For the generation of pLVX-*TMEM166* and *TMEM166-GFP*, *TMEM166* cDNA was amplified from the cDNA library of Huh7 cells by PCR and cloned into pLVX-puro and pEGFP-N1, respectively.

For the generation of *GFP-ACSL3* and *mcherry-ACSL3*, *ACSL3* cDNA was amplified from the cDNA library of Huh7 cells by PCR and cloned into pEGFP-C1 and pmcherry-C1, respectively.

### Cell culture and cell lines construction

HEK293T, L02, Huh7 and BEL-7402 cell lines without mycoplasma contamination were cultured in DMEM (Gibco, C11995500BT) supplemented with 10% fetal bovine serum (HUANKE, HK-CH500) and maintained at 37 °C in a humidified chamber containing 5% CO2.

*TMEM166* KO cell line was established in Huh7 cells by *CRISPR/Cas9*-mediated genome editing. The *TMEM166* genomic DNA was amplified by PCR and sequenced using the forward primer (5′- GTAGGCTGGAGTCATATTGG-3′) and reverse primer (5′-CACAATATTAGCAGAGCTGGG-3′). To generate *TMEM166* KO cell lines that stably expressing *TMEM166*, *shNC*, *shATF4*, *lentiCRISRR v2*^*_*^*sgCon*, *lentiCRISRR v2*^*_*^*sgXBP1s* and *lentiCRISRR v2*^*_*^*sgATF6*, *TMEM166* KO cells were transduced with the appropriate lentivirus and selected in the presence of 5 μg/mL puromycin.

### Quantitative real time PCR (qRT-PCR) assays

Total RNA samples were extracted with the TRIzol reagent, then equivalent RNA samples were then reverse-transcribed into cDNA using HiScript III 1st Strand cDNA Synthesis Kit (Vazyme, R312-02) according to the manufacturer’s protocol. All gene transcripts were quantified by real-time PCR with Taq Pro Universal SYBR qPCR Master Mix kit (Vazyme, Q712-03). The primers used for RT-qPCR were listed in Supplementary Table 2.

### Co-Immunoprecipitation and western blot analysis

The total protein from cells was extracted using RIPA Lysis Buffer (Beyotime, China; P0013B) containing proteinase inhibitor cocktail (Roche Diagnostics, Germany; 04693116001) and phosphatase inhibitor cocktail (Roche, 04906837001). Nuclear proteins were obtained from cells using the Nuclear Protein Extraction Kit (Solarbio, China; EX1470) following the manufacturer’s instructions. For Co-Immunoprecipitation, cell extracts (600 μL) were mixed with precleared protein G sepharose TM Fast Flow (GE Healthcare, 17-0618-01), then incubated with anti-GFP affinity beads 4FF (SA07005, Smart-Lifescience, China) overnight. The beads were collected by centrifugation, washed five times, resuspended in 2 × SDS loading buffer, and analyzed by western blotting.

For normal western blot analysis, protein concentrations were determined using a BCA protein assay reagent (Solarbio, China; PC0020). Equal amounts of proteins were separated by SDS-PAGE electrophoresis and transferred to polyvinylirdenediflouride (PVDF) membranes at 4 °C. After blocking with 5% nonfat milk for 1 h, the membranes were incubated with the primary antibodies overnight at 4 °C, and then incubated with HRP-labeled secondary antibodies. The protein bands were visualized using a chemiluminescence image analysis system (Invitrogen, iBright 750). The scanned bands were quantified using ImageJ software. The results were representative of at least three experiments.

### SUnSET assay

Cells were treated with puromycin (10mg/mL) for 10 min at 37°C after washing with ice-cold PBS, and then lysed for western blotting with anti-puromycin antibody. Quantify the incorporation of puromycin into nascent polypeptide to compare protein synthetic rate.

### Transmission electron microscope assay

The treated cells were fixed in a mixture of paraformaldehyde (2%) and glutaraldehyde (2.5%) for 1 h at 4°C. Then cells were embedded in Ultracut and sliced in 70 nm sections. Ultrathin sections were stained on-grid with 2% uranyl acetate (20 min) and lead citrate (5 min). Imaging was carried out using H-7650B transmission electron microscope (Hitachi, Tokyo, Japan).

### Immunofluorescence, thioflavin T (ThT) staining and confocal microscopy

For immunofluorescence staining, treated cells were fixed in 4% PFA (dissolved in PBS) for 10 min and permeabilized with 0.1% Triton X-100 for 15 min at room temperatures, then blocked with 5% BSA (dissolved in PBS) for 1 h. These cells were incubated with primary antibodies overnight at 4 °C, following stained with fluorescein-labeled secondary antibodies for 1 h. Nuclei were stained with Hoechst 33342 (0.5 μg/mL) for 10 min. After washing, the cells were observed and imaged under a confocal microscope (LSM 880 Meta plus Zeiss Axiovert zoom, Zeiss).

ThT staining was performed as described previously with some modification [51]. Briefly, the cells were fixed with 4% PFA for 30 min at room temperatures, then permeabilized with Permeabilizing Solution (0.5% Triton X-100, 3 mM EDTA) for 30 min on ice with gentle shake. After stained with 20 mM ThT in PBS for 30 min and Hoechst 33342 (0.5 μg/mL) for 10 min, cells were imaged by a LSM 880 confocal microscope.

### Cell viability and colony formation assays

Treated cells were seeded in 96-well plates (3 × 10^3^ cells/well; five replicates), serum-free starved for 24 h and then pulsed with 10% FCS for the indicated time. Cell viability assays were performed using the CCK8 Assay kit according to the manufacturer’s instructions. Cell viability was calculated as follows: cell viability = absorbance of test group/absorbance of control group × 100%. Each experiment was performed in biological triplicate and independently repeated three times.

For the colony formation assay, cells were plated in triplicate at 1000 cells/well and cultured for two weeks in 12-well plates. Then cells were fixed with 4% PFA (dissolved in PBS) for 10 min and stained with crystal violet for 30 min. Colonies were photographed and the total area covered by the attached colonies was quantified using the ImageJ software.

### Oxygen consumption rate (OCR) and fatty acid β-oxidation (FAO) analysis

For OCR measurement, 10^4^ cells were seeded in 96-well XF plates two days before the measurement. Following hydrating an Agilent Seahorse XF96 sensor cartridge with 1 mL of XF calibrant in a non-CO2 37°C incubator overnight, oxygen consumption rate was measured in a Seahorse XF24 extracellular flux analyzer according to the manufacturer’s instruction with 1.5 μM oligomycin, 1 μM FCCP, 0.5 μM Rotenone and 0.5 μM antimycin A.

For FAO measurement, 10^4^ cells were seeded at each well in 96-well XF plates in full-growth medium three days before the measurement. The growth medium was changed to substrate-limited medium (DMEM, 0.5 mM glucose, 1 mM GlutaMAX, 1% FBS and 0.5 mM carnitine) one day before the experiment and an Agilent Seahorse XF96 sensor cartridge was hydrated with 1 mL of XF calibrant in a non-CO2 37°C incubator overnight. Oxygen consumption rate was measured in a Seahorse XF24 extracellular flux analyzer according to the manufacturer’s instruction with 1.5 μM oligomycin, 2 μM FCCP, 0.5 μM Rotenone and 0.5 μM antimycin A following the addition of BAS or PA.

### Tissue samples and immunohistochemistry (IHC) analysis

Tissue samples (including HCC tissues and para-cancer tissues) were collected from Shandong Medical College Affiliated Linyi People’ Hospital (Linyi, China). These tissue samples were preserved through paraffin embedding. For the IHC analysis, paraffin-embedded samples were sectioned and subjected to a series of procedures, including dewaxing, rehydration with gradient ethanol, antigen retrieval, treatment with a 3% hydrogen peroxide solution to block endogenous peroxidase activity, and thorough washing with PBS. The slides were then incubated in 5% goat serum. Following incubation with primary antibodies at 4 °C overnight and washing three times in PBS, the sections were incubated with secondary antibodies, and the final antigen detection was performed using an IHC kit (Gene Tech, GK600705). This retrospective study was approved by the Science and Technology Ethics Committee of Shandong Medical College Affiliated Linyi People’ Hospital (No. 202506-H-004), and was performed in accordance with the approved guidelines.

### Statistical analysis

Statistical analyzes were carried out with GraphPad Prism 9 (GraphPad, La Jolla, CA, USA). Statistical comparisons between two groups were carried out using Student’s t-test, while statistical comparisons among multiple groups were carried out using One-way ANOVA. The differences between groups were statistically significant when *P* < 0.05. Data are presented as the mean ± SD from at least three independent experiments.

## Supplementary information

Supplementary Figures and Figure Legends

Supplementary Table 1

Supplementary Table 2

Original Western Blot

## Data Availability

All data generated or analyzed in this study are included in the published article or supplementary materials. The data can be made available upon reasonable request to the corresponding author.
