# Supplementary material for: TMEM166 negatively regulates unfolded protein response to affect hepatocellular carcinoma cell growth and sorafenib resistance
Source: Cell Death Dis. 2025 Nov 5;16(1):794. doi: 10.1038/s41419-025-08176-w (PMC12589423; doi:10.1038/s41419-025-08176-w)
Supplement: Supplementary file 1 — Supplementary Figures and Figure Legends [file 41419_2025_8176_MOESM1_ESM.pdf]

Supplementary Figures and Figure Legends

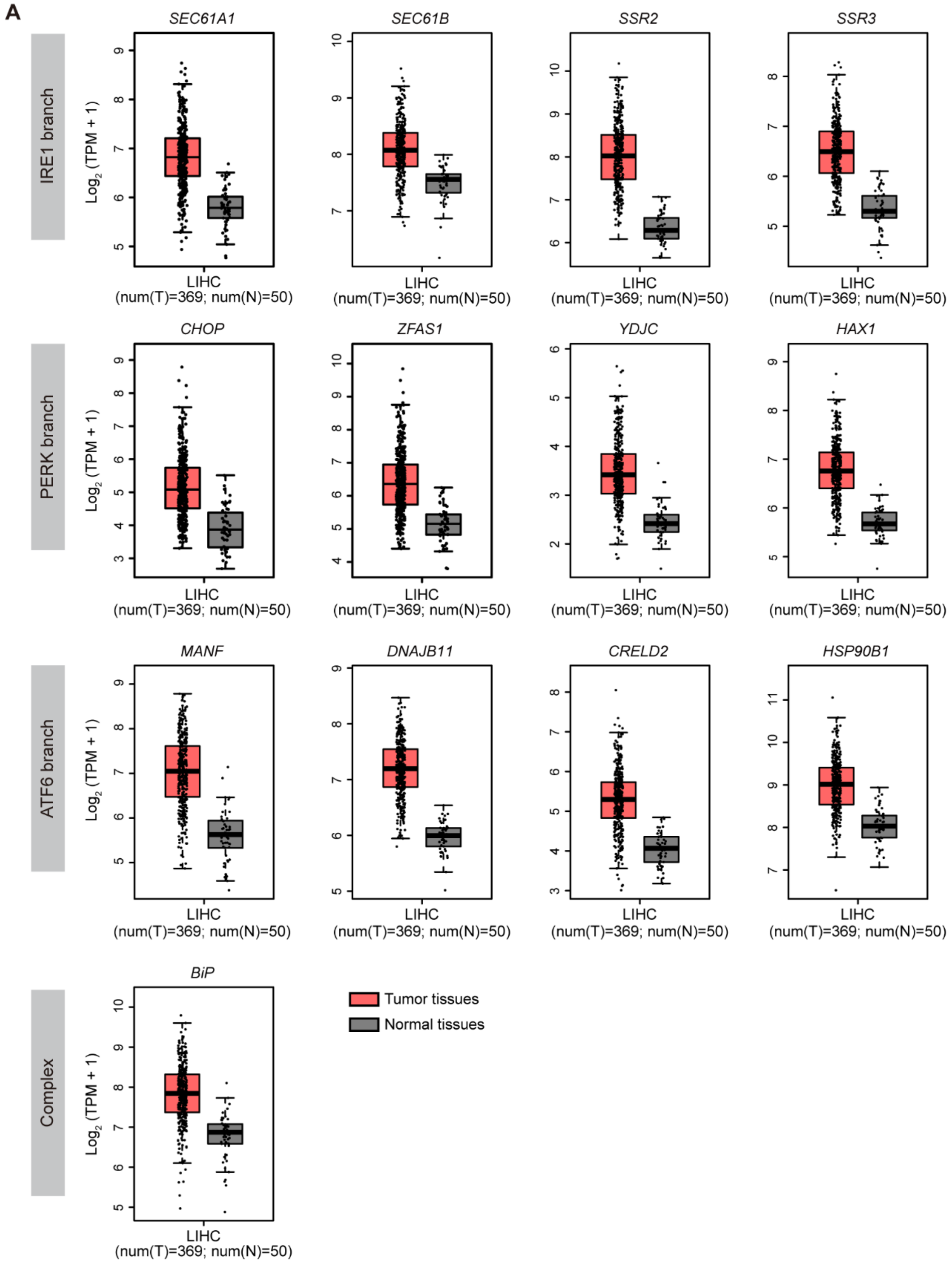

Fig. S1 The UPR activity in HCC and normal liver tissues. Differential expression analysis for UPR-targeting genes in HCC and normal tissues. Data are obtained from TCGA and analyzed by using the GEPIA 2.

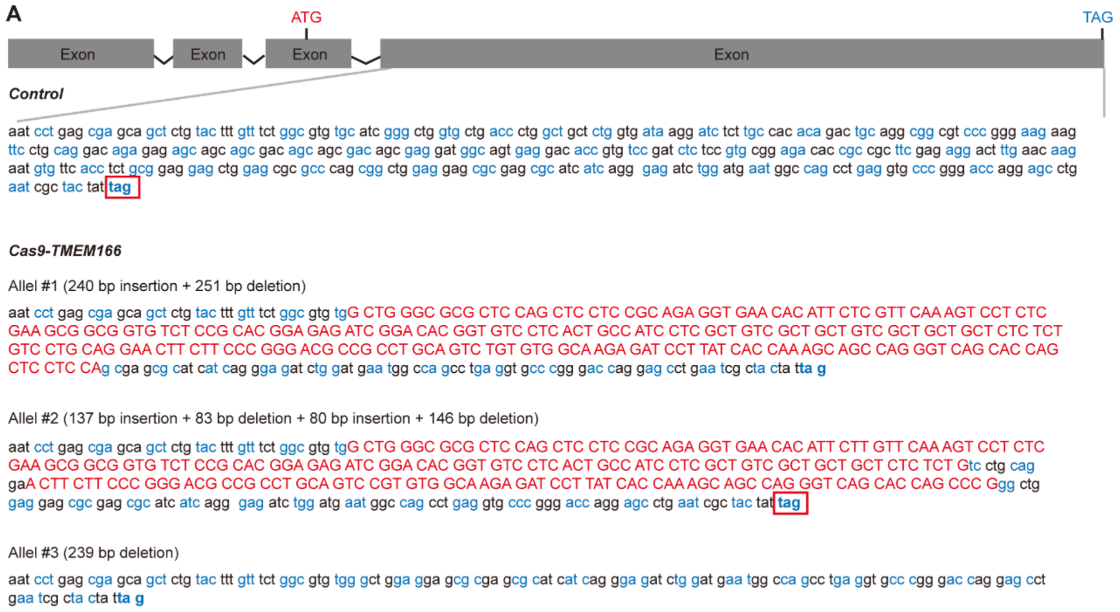

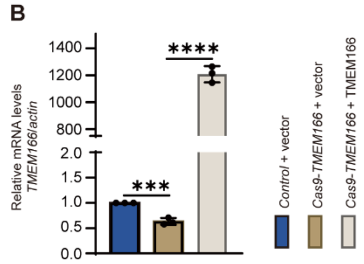

Fig. S2 Validation of Cas9-*TMEM166*/Huh7 cells and Cas9-*TMEM166*/Huh7 overexpressing TMEM166 (**A**) Genotyping of Cas9-*TMEM166*/Huh7 cells. (**B**) The levels of *TMEM166* mRNA in in indicated cells were detected by qRT-PCR. ***P<0.001, ****P<0.0001. Data (mean ± SD) are representative of at least three independent experiments.

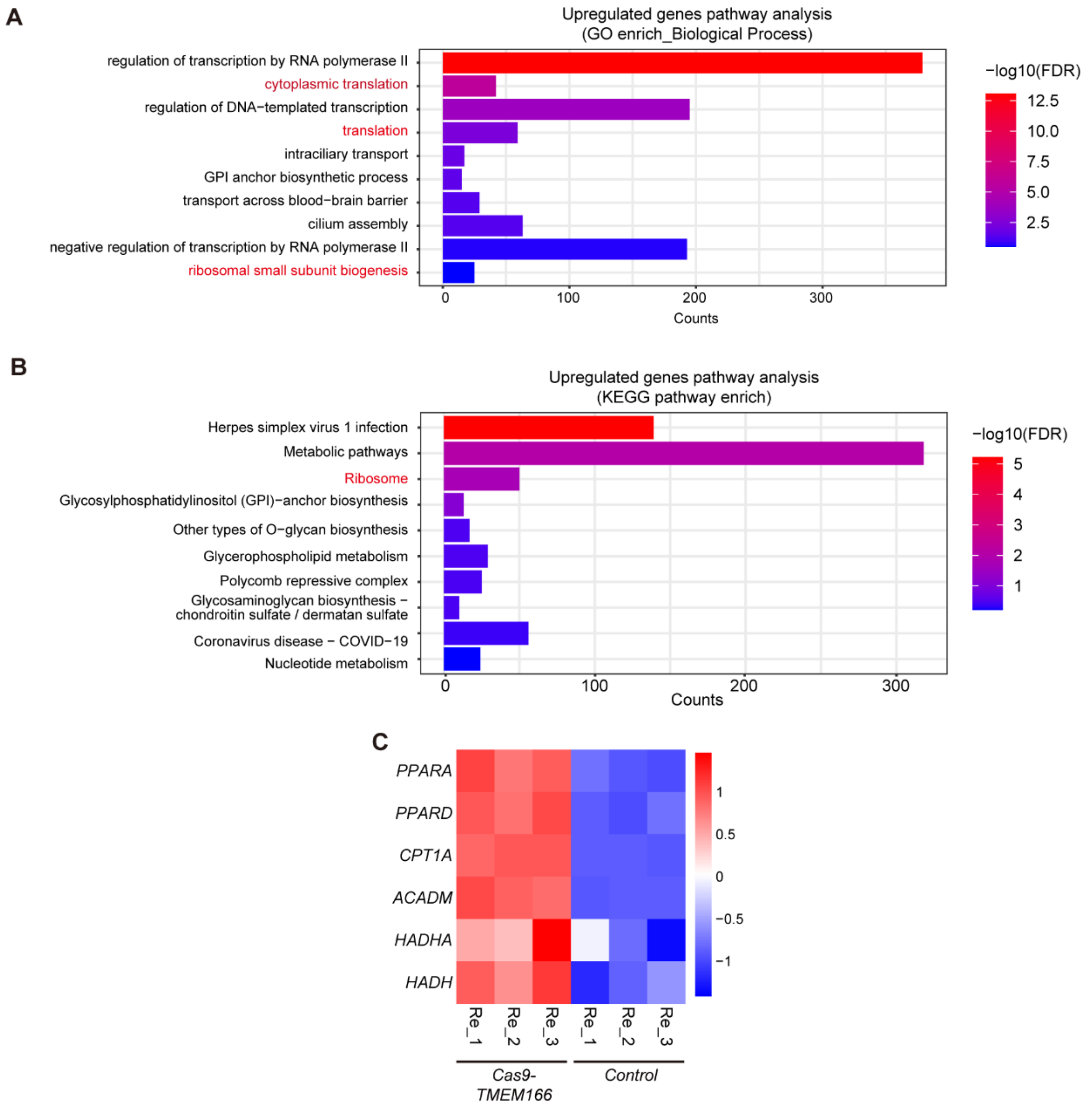

Fig. S3 Analysis of the data of RNA-Seq. (**A and B**) GO or KEGG enrichment analysis of upregulated genes in Cas9-*TMEM166*/Huh7 cells compared with *Control*/Huh7 cells from the data of RNA-Seq. (**C**) Heatmap of the expression levels of FAO-related genes in *Control* and Cas9-*TMEM166*/Huh7 cells obtained from RNA-Seq.

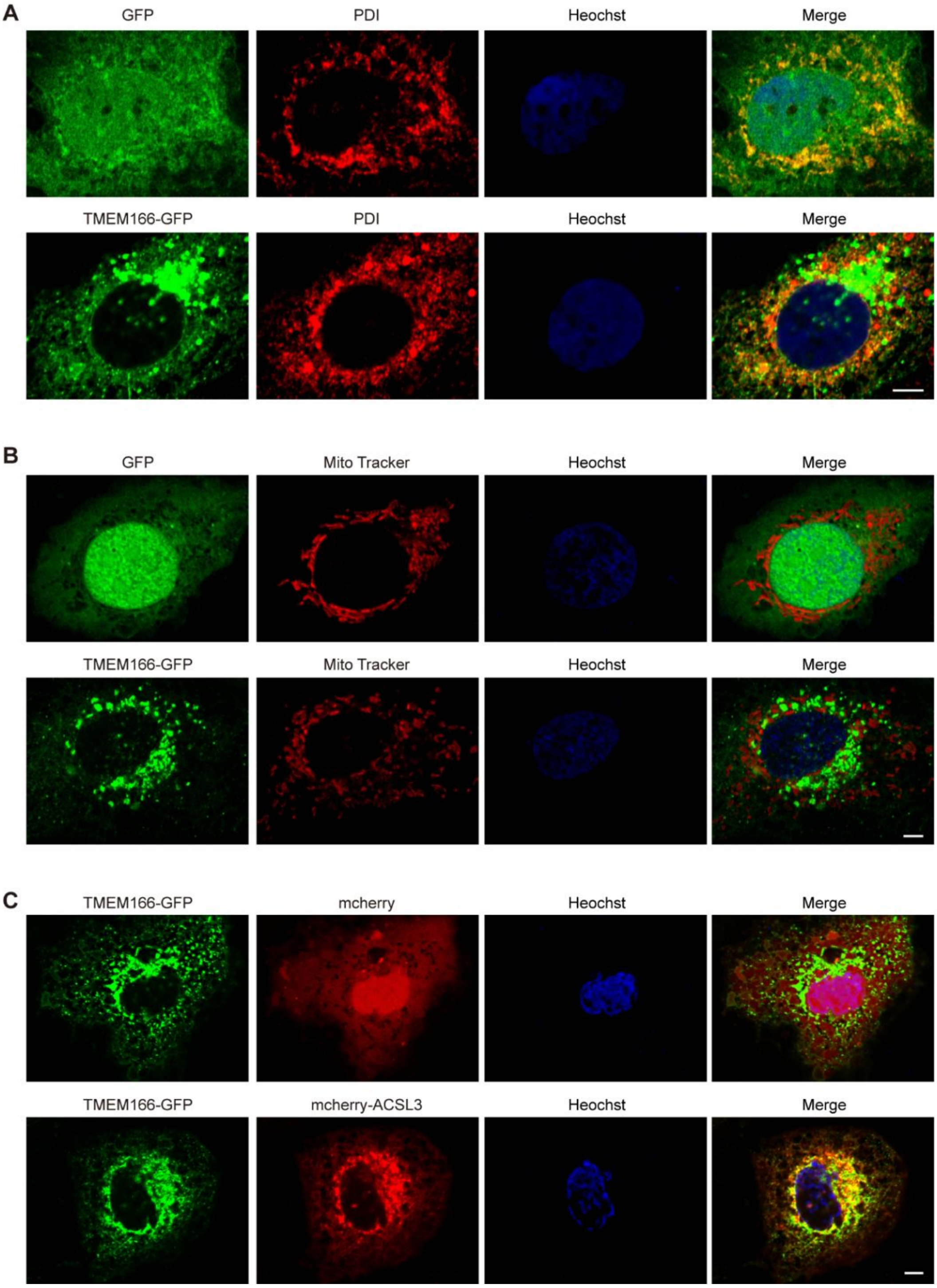

Fig. S4 TMEM166 localizes in ER, and colocalizes with ACSL3. (A-B) Huh7 cells were transfected with indicated plasmids for 24 h and stained with anti-PDI antibody (A) or Mito-Tracker (100 nM, 15 min) (B), and then observed by confocal microscope. Representative fluorescence images were shown. Scale bars, 5 μm. **(C)** Huh7 cells were transfected with indicated plasmids for 24 h and observed by confocal microscope. Representative fluorescence images were shown. Scale bars, 5 μm.

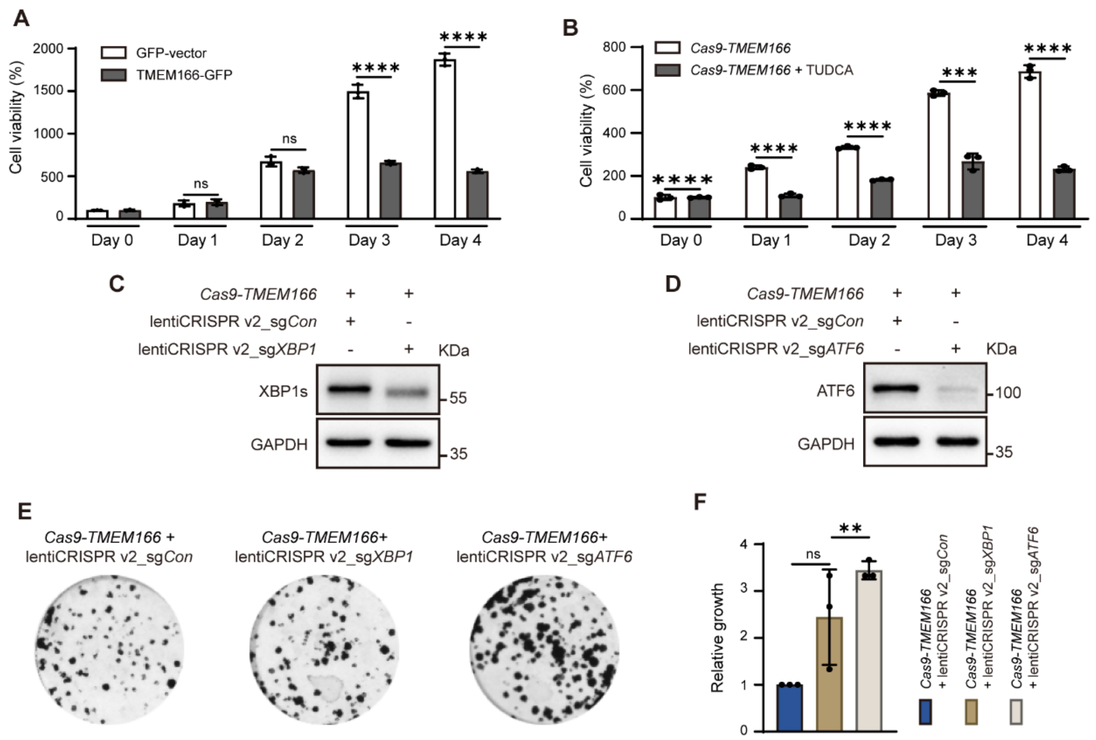

Fig. S5 Loss of *TMEM166* promotes Huh7 cell proliferation independent with XBP1 and ATF6. (**A**) BEL-7402 cells were seeded in 96-well plates (1.5×10^3 cells/well; 3 replicates) and transfected with vector-GFP or TMEM166-GFP for indicated time. Cell viability was detected by CCK-8 assay. (**B**) Cas9-*TMEM166*/Huh7 cells were serum starved for 24 h and then seeded in 96-well plates (1.5×10^3 cells/well; 3 replicates), pulsed with 10% FBS and treated with or without TUDCA (200 μM) for indicated time. Cell viability was detected by CCK-8 assay. (**C and D**) The levels of XBP1s and ATF6 were detected by immunoblotting in indicated cells. (**E**) Detection of clonal formation in indicated cells. Representative images of colony formation were shown. (**F**) Relative area of clones. Average value in *lentiCRISPR* v2_sg*Con* + expressing Cas9-*TMEM166*/Huh7 cells was normalized as 1. ***P*<0.01, ****P*<0.001, *****P*<0.0001, ns, no significant. Data (mean ± SD) are representative of at least three independent experiments.

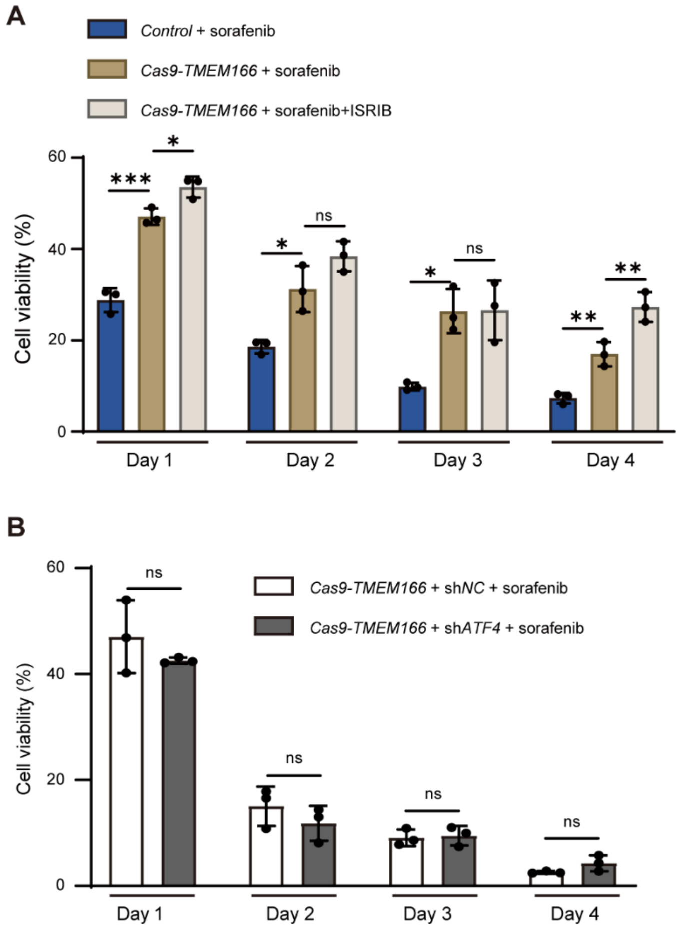

Fig. S6 *TMEM166* deletion increases sorafenib resistant independent with ATF4 branch in Huh7 cells. (**A**) *Control* and Cas9-*TMEM166*/Huh7 cells were seeded in 96-well plates (3 × 10^3 cells/well; 3 replicates) and treated with sorafenib (5 μM), and with or without ISRIB (1 μM) for indicated time. Cell viability was detected by CCK-8 assay. (**B**) The indicated cells were seeded in 96-well plates (3 × 10^3 cells/well; 3 replicates) and treated with sorafenib (5 μM) for different time. Cell viability was detected by CCK-8 assay. **P*<0.05, ***P*<0.01, ****P*<0.001, ns, no significant. Data (mean ± SD) are representative of at least three independent experiments.
