# Supplementary material for: TMEM166 negatively regulates unfolded protein response to affect hepatocellular carcinoma cell growth and sorafenib resistance
Source: Cell Death Dis. 2025 Nov 5;16(1):794. doi: 10.1038/s41419-025-08176-w (PMC12589423; doi:10.1038/s41419-025-08176-w)
Supplement: Supplementary file 2 — Supplementary Table 1 [file 41419_2025_8176_MOESM2_ESM.docx]

**Supplementary Table1 List of antibodies and reagents used in this manuscript**

| **Antibodtes and regeants** | **Art. No of the Products** | **Company** |
| --- | --- | --- |
| Anti-TMEM166 | GTX32925 | GeneTex |
| Anti-ATF4 (IHC) | ab31390 | Abcam |
| Anti-pIRE1 | ab124945 |  |
| Anti-ATF6 | ab83504 |  |
| Anti-PDI | ab2792 |  |
| Anti-XBP1s (IHC) | 647501 | Biolegend |
| Anti-IRE1 | 3294S | Cell Signaling Technology |
| Anti-XBP1s (WB) | 12782S |  |
| Anti-PERK | 3192S |  |
| Anti-ATF4 (WB) | 11815 |  |
| Anti-pAMPK | 2535S |  |
| Anti-AMPK | 2532S |  |
| Anti-P-p70 S6 kinase | 9234S |  |
| Anti-p70 S6 kinase | 2708S |  |
| Anti-ACSL3 | 83319T |  |
| Goat anti-Mouse IgG (H+L) Secondary Antibody, HRP | 31430 | Invitrogen |
| Goat anti-Rabbit IgG (H+L) Secondary Antibody, HRP | 31460 |  |
| Anti-GFP | KM8009 | Tianjin Sungene Biotech |
| Anti-GAPDH | KM8002 |  |
| Anti-Flag | F3040 | Sigma |
| Anti-Histone 3 | P01L24 | Gene-Protein Link |
| Anti-puromycin | MABE343 | Merck |
| Tunicamycin (Tm) | ab120296 | Abcam |
| Oligomycin | ab141829 |  |
| FCCP | M9051 | AbMole |
| Rotenone | M6209 |  |
| Hoechst33342 | H1399 | Invitrogen |
| MitoTrackerTM Red CMXRos | M7512 |  |
| TRIzol reagent | 15596-026 |  |
| Thapsigargin (Tg) | T9033 | Sigma |
| Thioflavin T (ThT) | T3516-5G |  |
| BSA | A6003-5G |  |
| Sodium palmitate | P9767-5G |  |
| Oil red O staining kit | G1262 | Solarbio |
| CCK-8 Assay Kit | CA1210 |  |
| L-carnitine | S2388 |  |
| Sorafenib | S7397 | Selleck |
| MCK8866 | S8875 |  |
| Triglyceride (TG) enzyme assay kit | E1013 | Applygen |
| NEOFECT^TM^ DNA transfection reagent | TF201201 | Bioss Inc. Neofect (beijing) biotech |
| ATP Assay Kit | S0026 | Beyotime |
| Opti-MEM | 31985-047 | Gibco |
| Antimycin A | GC49360 | GlpBio |
| Anti-GFP-Affinity-Beads | SA070001 | Smart-Lifesciences |
| crystal violet | E607309-0100 | Sangon Biotech |
| Trans-ISRIB | 5284/10 | Tocris Bioscience |
| TUDCA | T2532 | TargetMol |
