# Supplementary material for: TMEM166 negatively regulates unfolded protein response to affect hepatocellular carcinoma cell growth and sorafenib resistance
Source: Cell Death Dis. 2025 Nov 5;16(1):794. doi: 10.1038/s41419-025-08176-w (PMC12589423; doi:10.1038/s41419-025-08176-w)
Supplement: Supplementary file 3 — Supplementary Table 2 [file 41419_2025_8176_MOESM3_ESM.docx]

## Supplementary Table 2. The sgRNA, shRNA and Primer sequences used in this study

| **Name** | | **Sequence** |
| --- | --- | --- |
| Control shRNA | | 5'-CCTAAGGTTAAGTCGCCCTCG-3' |
| *ATF4* shRNA | | 5'-CCTAGGTCTCTTAGATGATTA-3' |
| Control sgRNA | | 5'-ACGGAGGCTAAGCGTCGCAA-3' |
| *TMEM166* sgRNA | | 5'-TTTGTTTCTGGCGTGTGCAT-3' |
| *XBP1* sgRNA | | 5'-CTTTAGGGGTCCCGTCGGCC-3' |
| *ATF6* sgRNA | | 5'-CGGGCTAAAAGGTGACTCCA-3' |
| *ACSL3* sgRNA | | 5'-CGAGTGGATGATAGCTGCAC-3' |
| **qRT-PCR** |  |  |
| *TMEM166* | Forward Primer | 5'-GCAACATCCTAGCGGCCTAT-3' |
|  | Reverse Primer | 5'-TGCAGTCTGTGTGGCAAGAG-3' |
| *XBP1s* | Forward Primer | 5'-TGAGTCCGCAGCAGGTGCA-3' |
|  | Reverse Primer | 5'-CTGGGTCCTTCTGGGTAGACCTC-3' |
| *XBP1u* | Forward Primer | 5'-GTTAAGACAGCGCTTGGGGA-3' |
|  | Reverse Primer | 5'-TGCACGTAGTCTGAGTGCTG-3' |
| *CHOP* | Forward Primer | 5'-GGAAACACAGTGGTCATTCCC-3' |
|  | Reverse Primer | 5'-CTGCTTGAGCCGTTCATTCTC-3' |
| *MANF* | Forward Primer | 5'-TTTACCAGGACCTCAAAGACAGA-3' |
|  | Reverse Primer | 5'-TTGCTTCCCGGCAGAACTTTA-3' |
| *BiP* | Forward Primer | 5'-CACAGTGGTGCCTACCAAGA-3' |
|  | Reverse Primer | 5'-TGTCTTTTGTCAGGGGTCTTT-3' |
| *Actin* | Forward Primer | 5'-TCATTCCAAATATGAGATGCGTTGT-3' |
|  | Reverse Primer | 5'-GCTATCACCTCCCCTGTGTG-3' |
| *PPARA* | Forward Primer | 5'-ATGGTGGACACGGAAAGCC-3' |
|  | Reverse Primer | 5'-CGATGGATTGCGAAATCTCTTGG-3' |
| *PPARD* | Forward Primer | 5'-CAGGGCTGACTGCAAACGA-3' |
|  | Reverse Primer | 5'-CTGCCACAATGTCTCGATGTC-3' |
| *CPT1A* | Forward Primer | 5'-ATCAATCGGACTCTGGAAACGG-3' |
|  | Reverse Primer | 5'-TCAGGGAGTAGCGCATGGT-3' |
| *ACADM* | Forward Primer | 5'-ACAGGGGTTCAGACTGCTATT-3' |
|  | Reverse Primer | 5'-TCCTCCGTTGGTTATCCACAT-3' |
| HADHA | Forward Primer | 5'-CTGCCCAAAATGGTGGGTGT-3' |
|  | Reverse Primer | 5'-GGAGGTTTTAGTCCTGGTCCC-3' |
| HADH | Forward Primer | 5'-CACACAGTAGTGTTGGTAGACC-3' |
|  | Reverse Primer | 5'-TGCCACTTTCCTAAGGCTTTC-3' |
