# Supplementary material for: TMEM166 negatively regulates unfolded protein response to affect hepatocellular carcinoma cell growth and sorafenib resistance
Source: Cell Death Dis. 2025 Nov 5;16(1):794. doi: 10.1038/s41419-025-08176-w (PMC12589423; doi:10.1038/s41419-025-08176-w)

Original data from Western Blot

Figure 2A

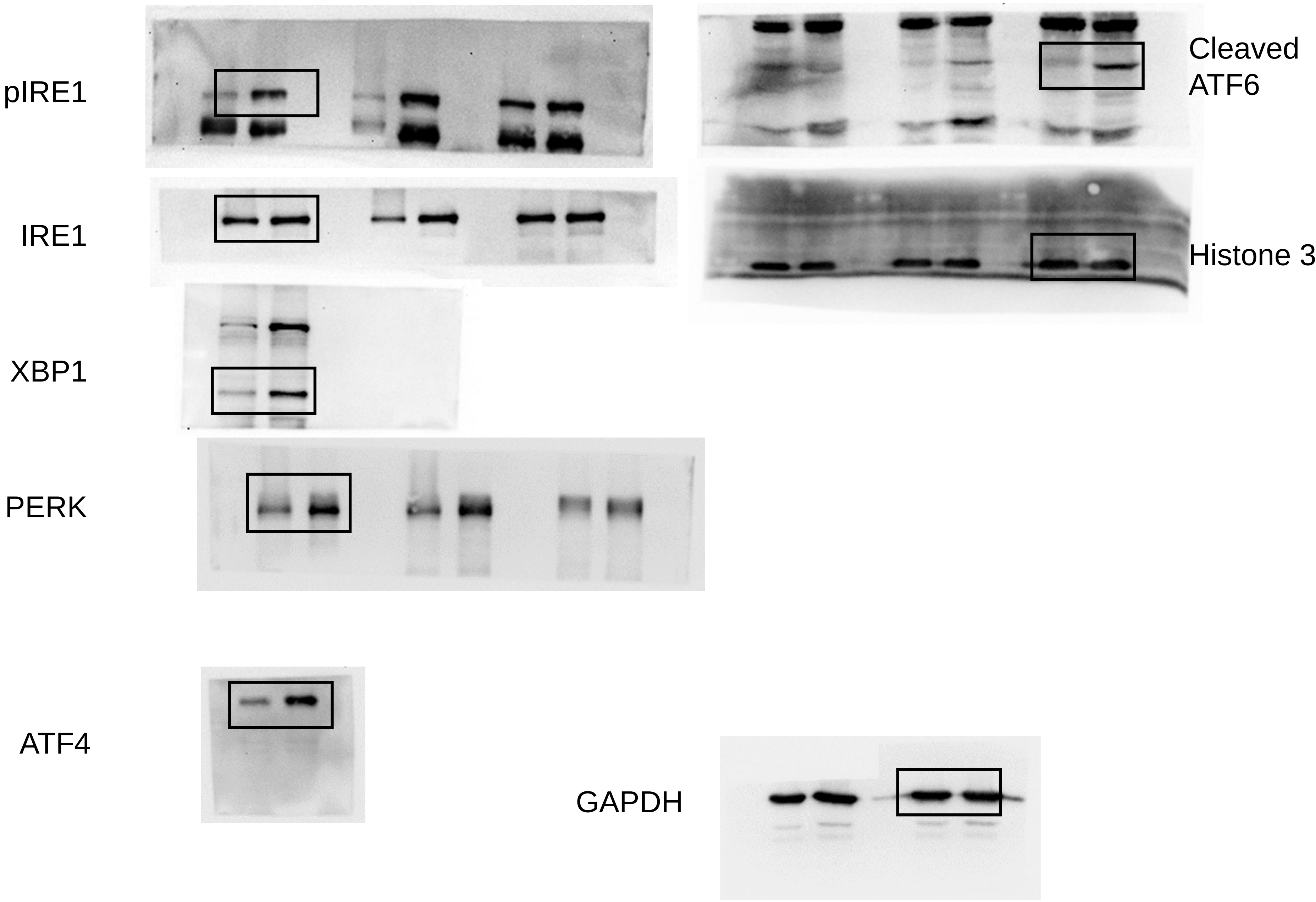

Figure 2D

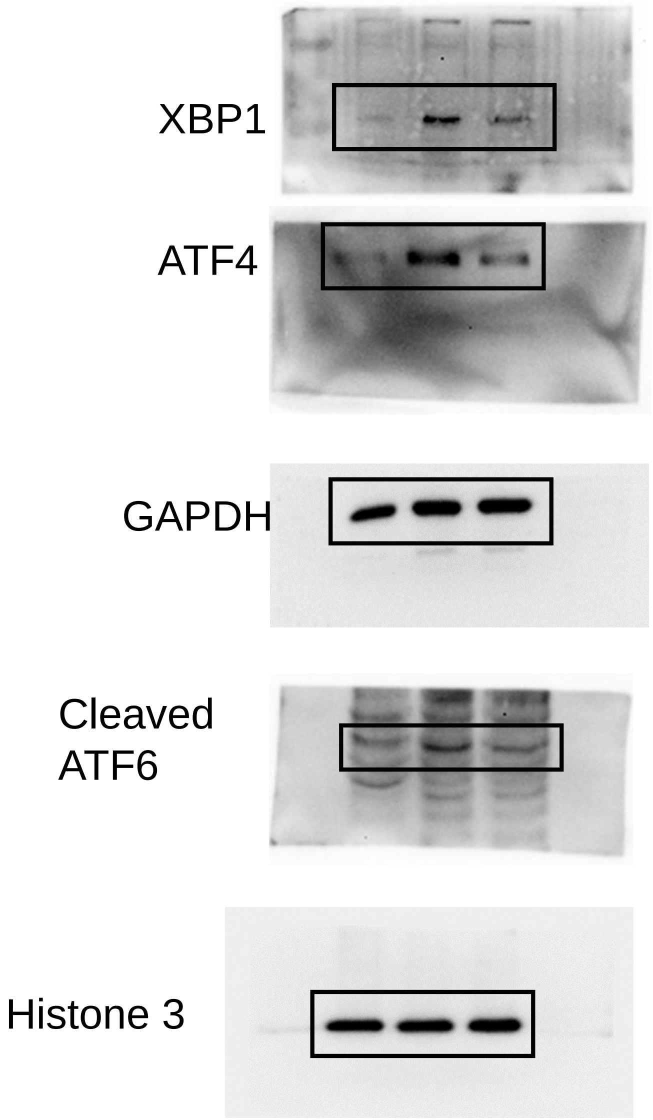

Figure 2E

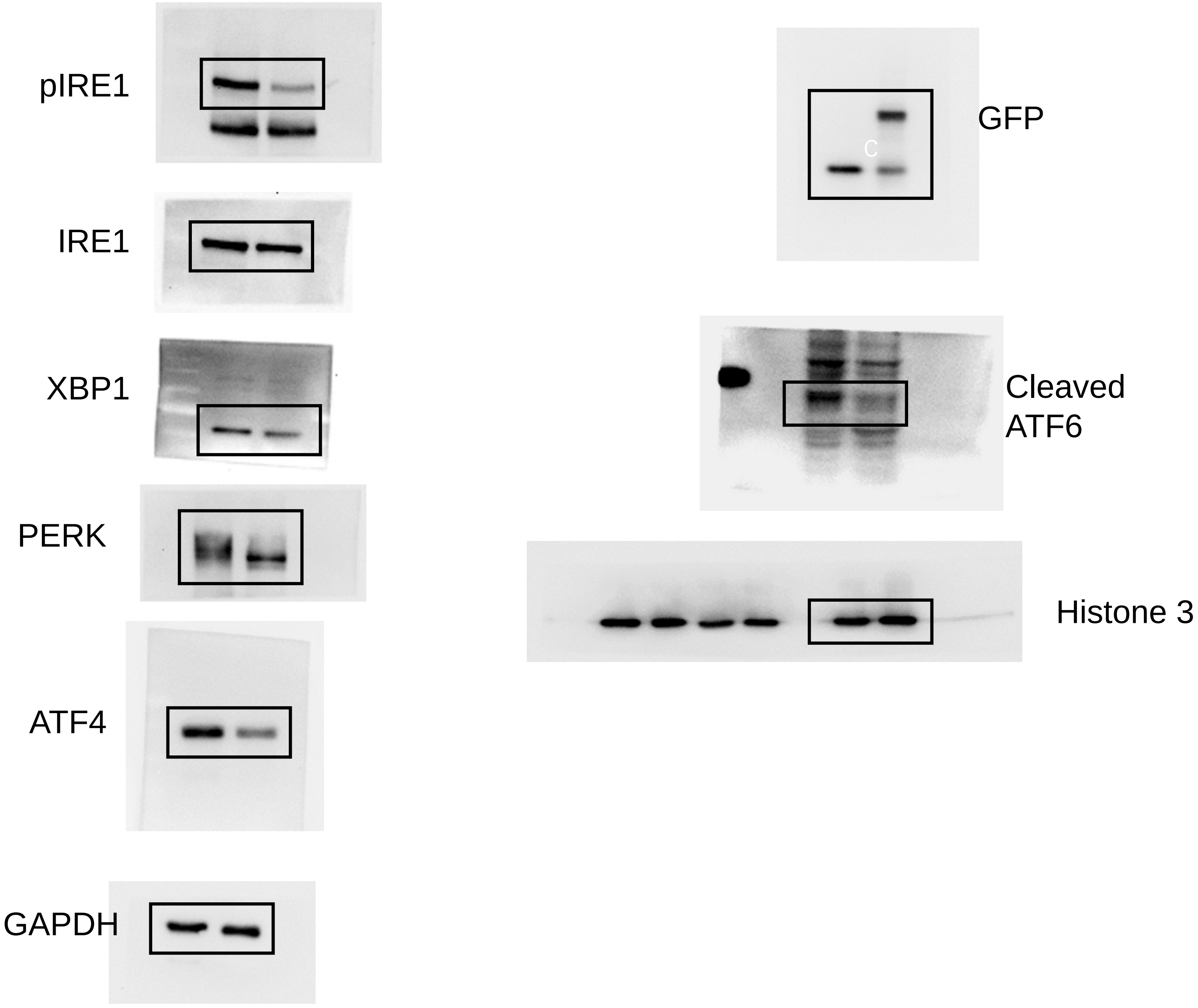

Figure 3E

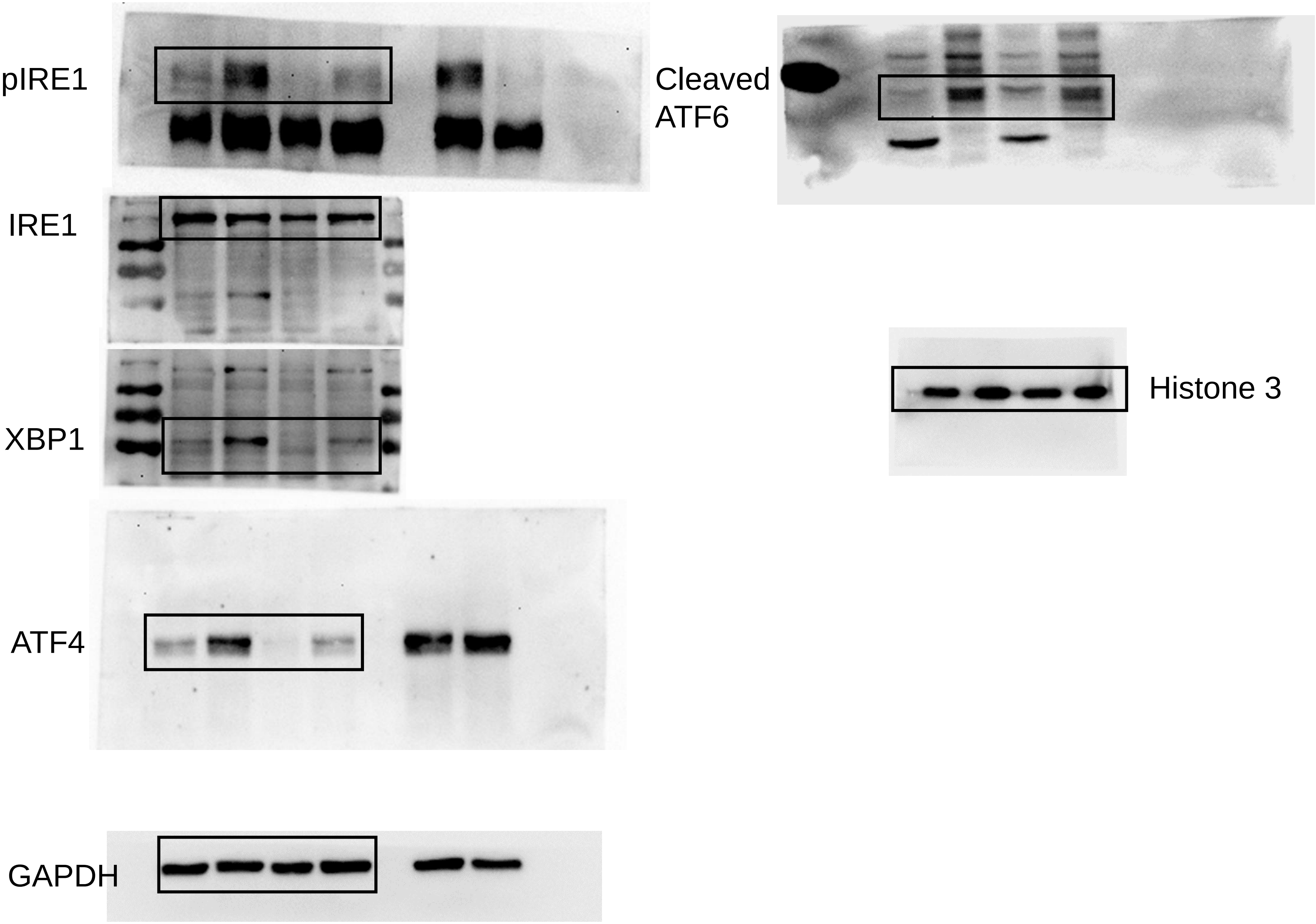

Figure 3F

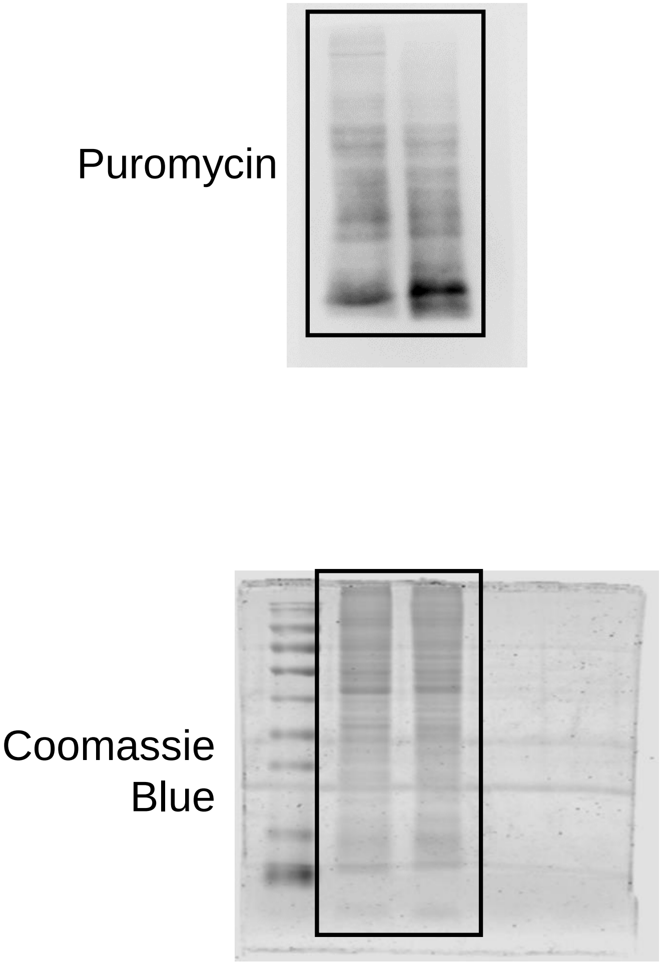

Figure 3H

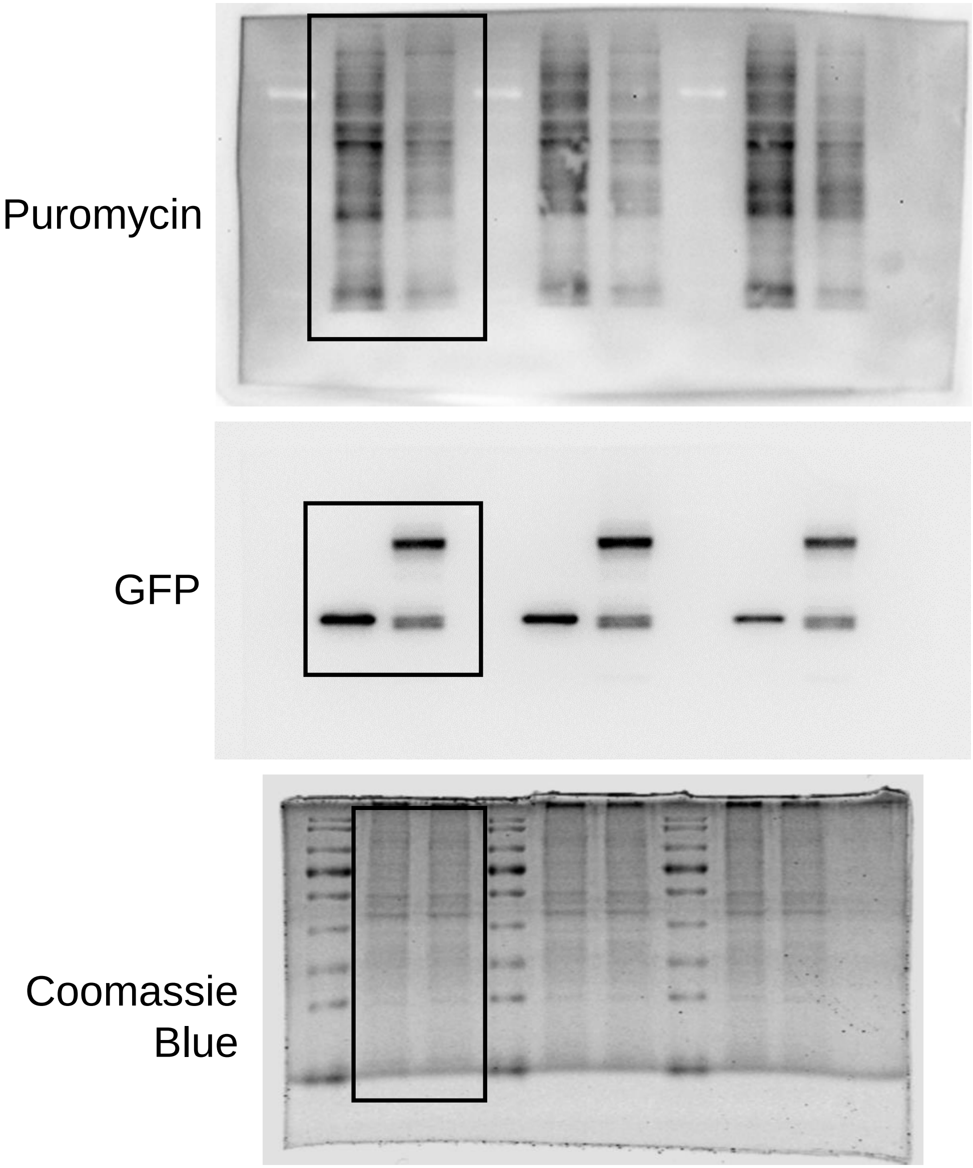

Figure 3J

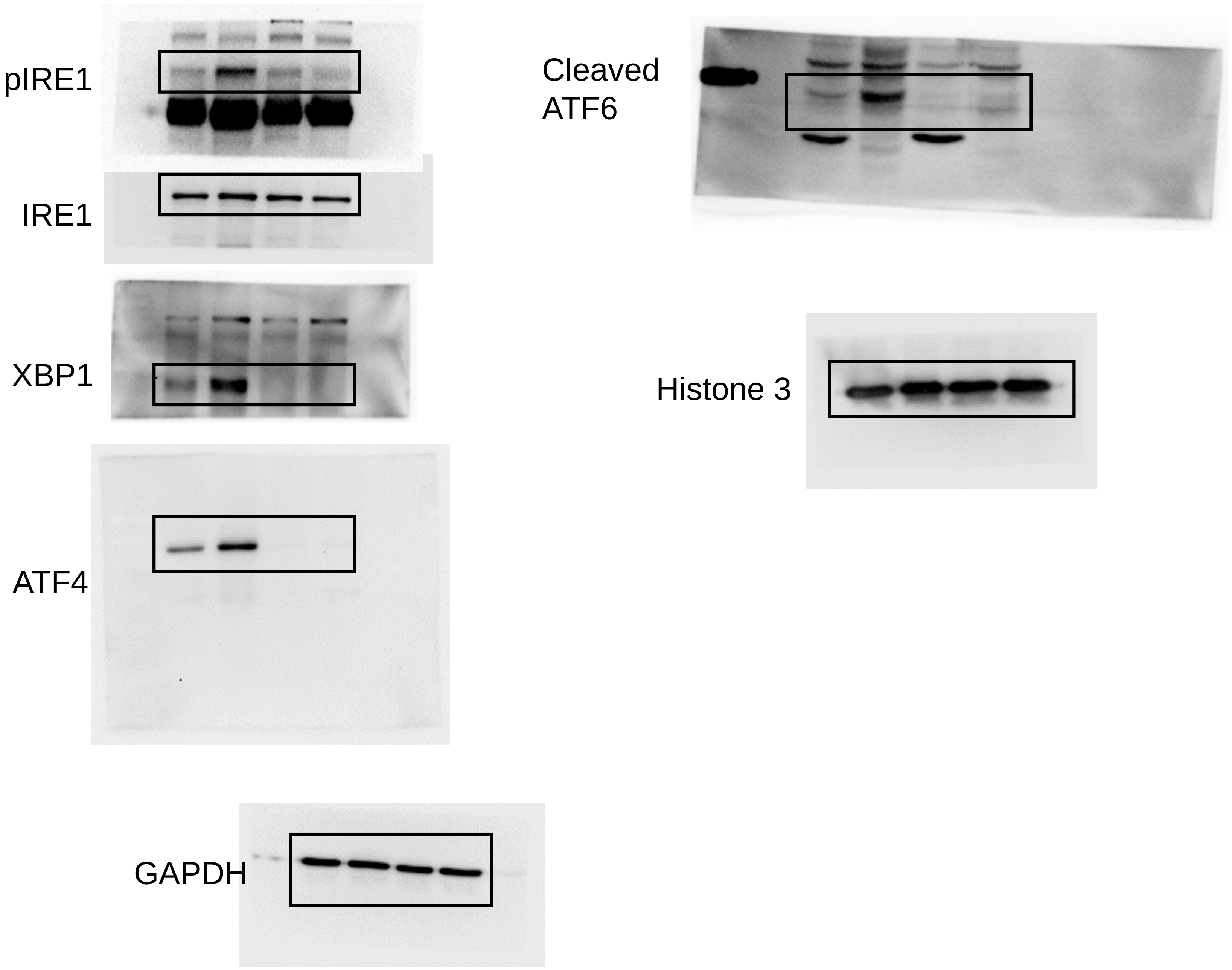

Figure 3K

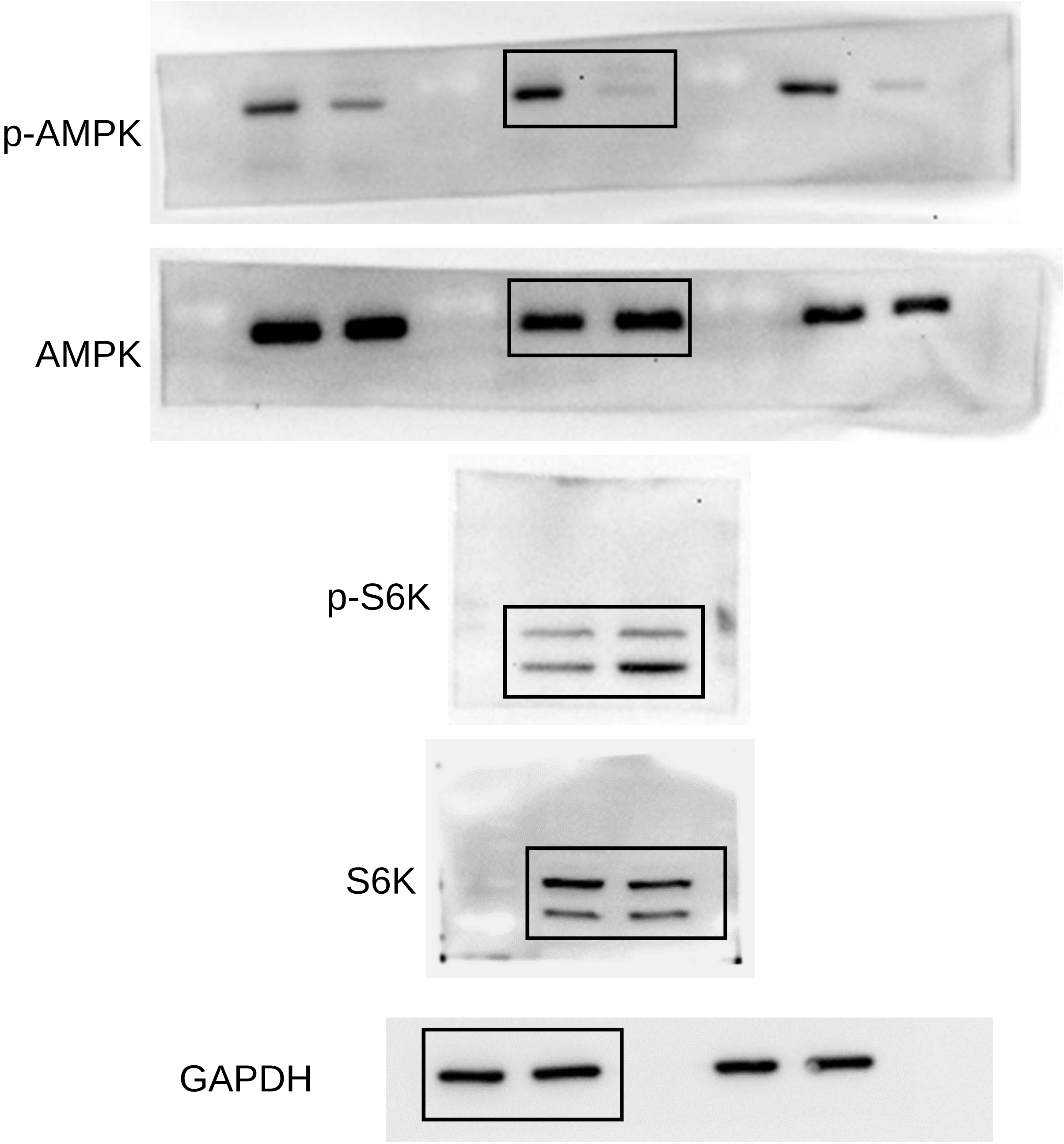

Figure 3M

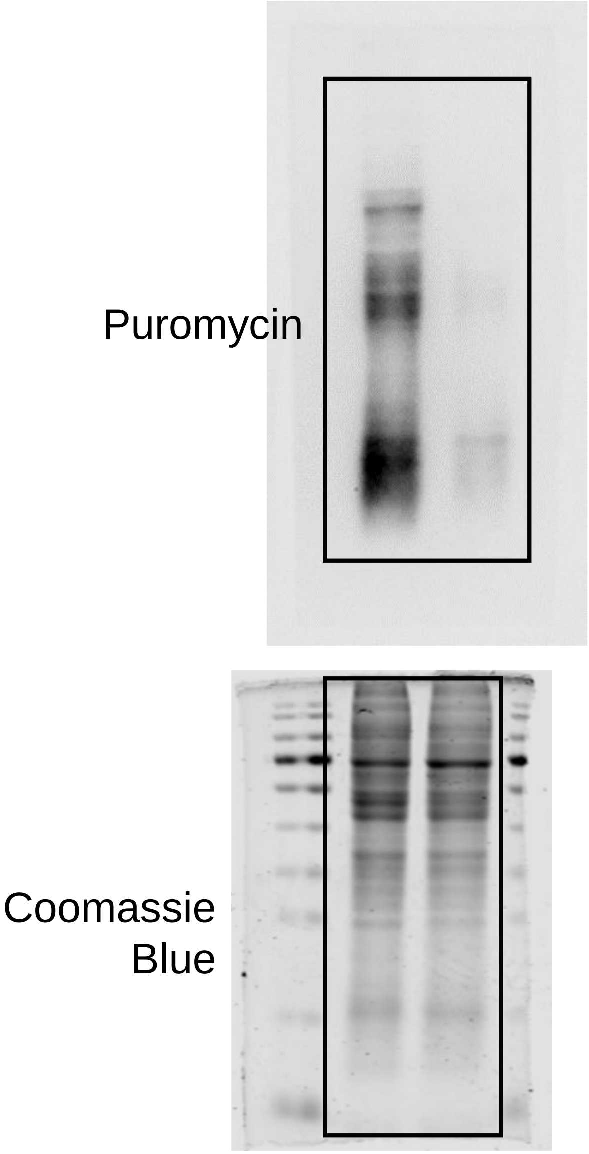

Figure 3N

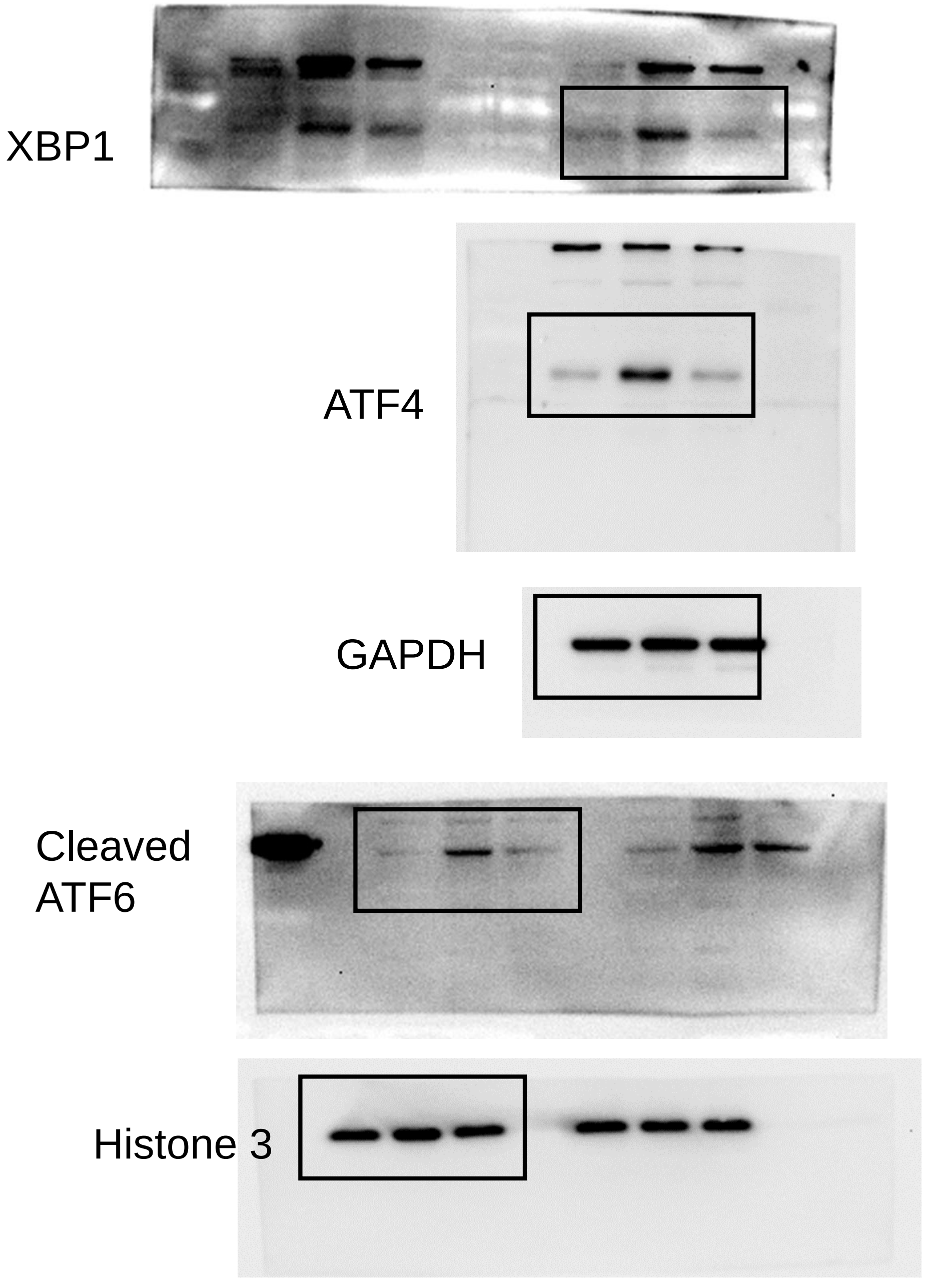

Figure 5A

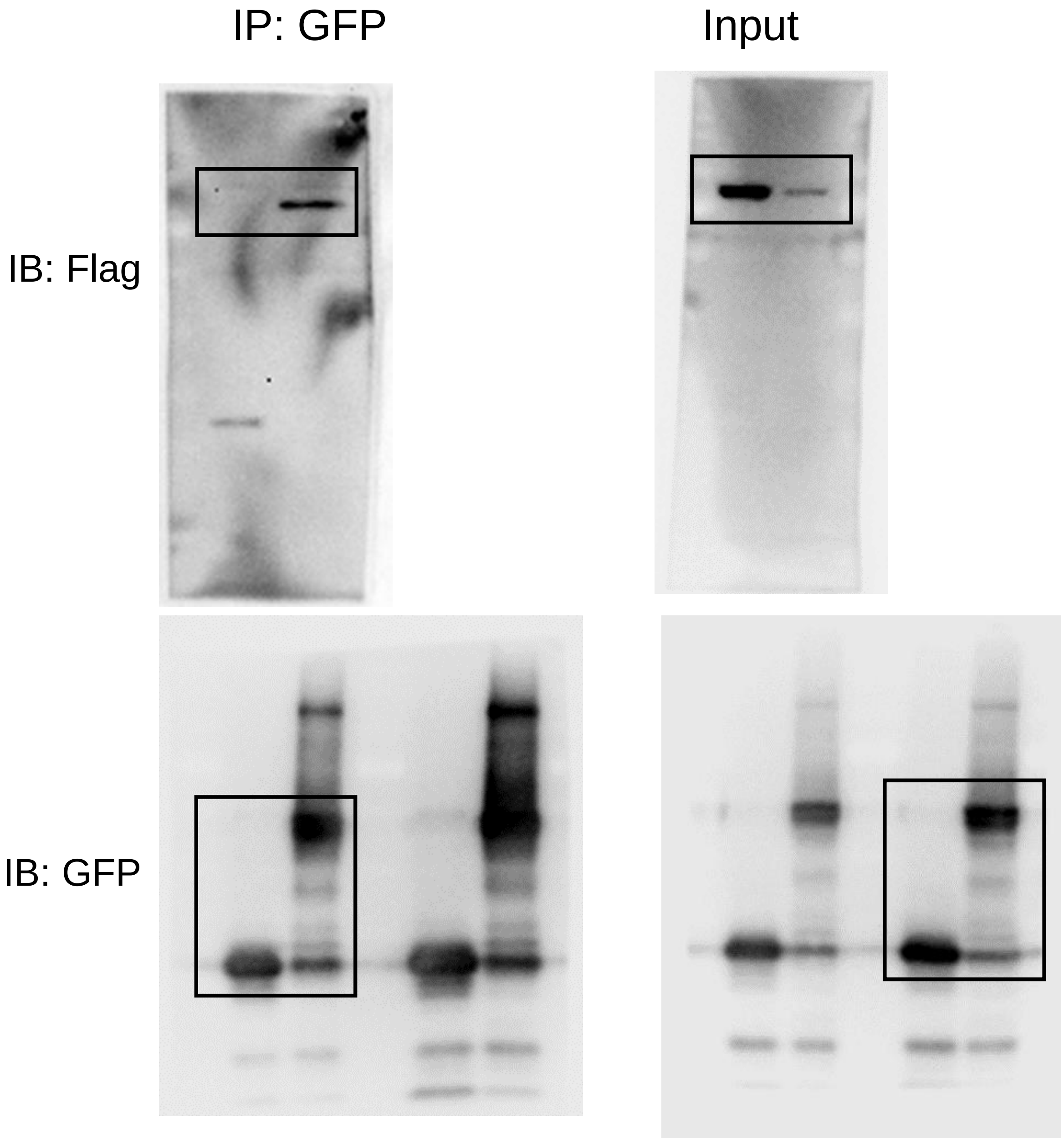

Figure 5B

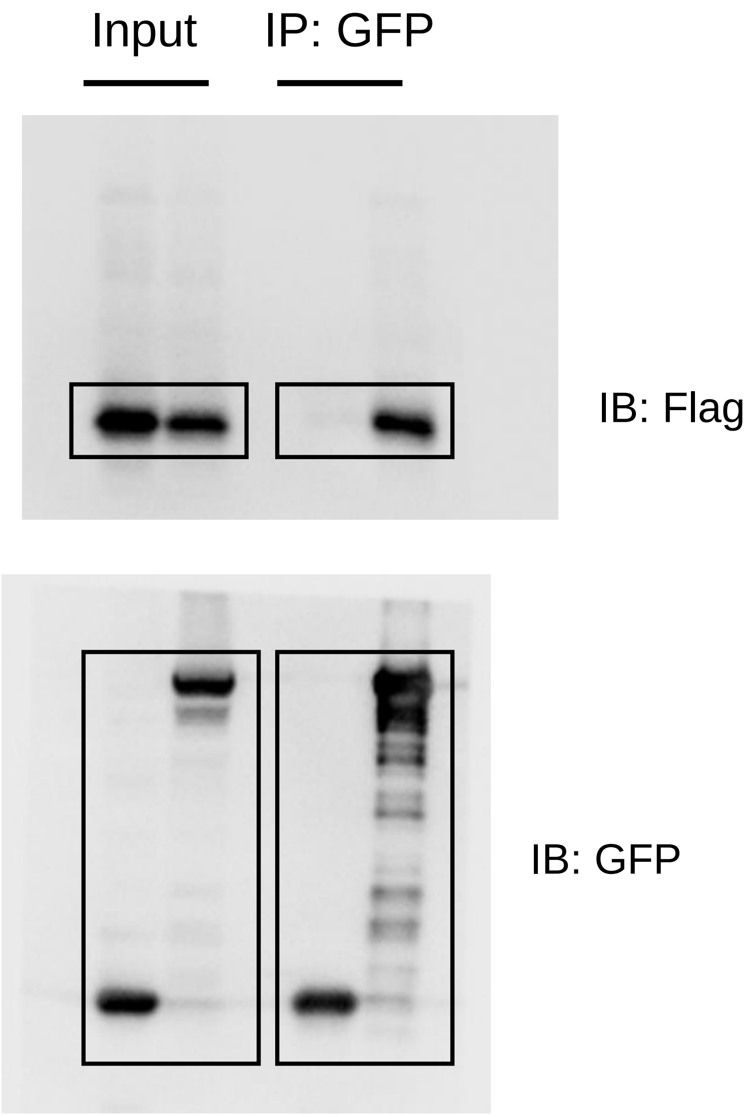

Figure 5C

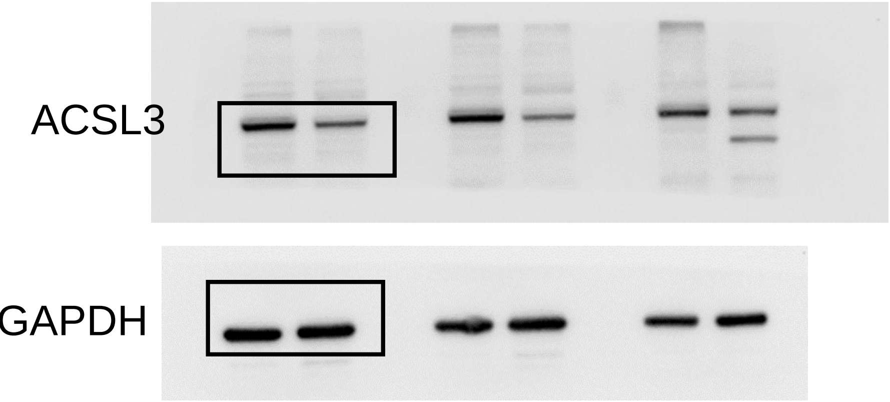

Figure 5E

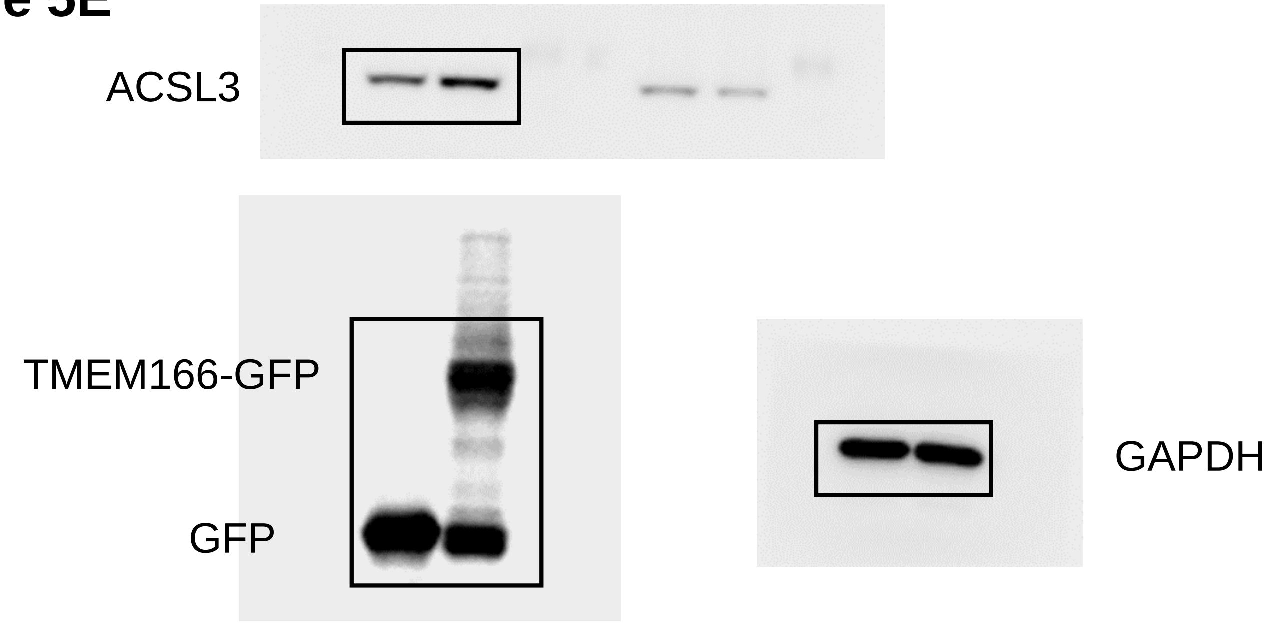

Figure 5G

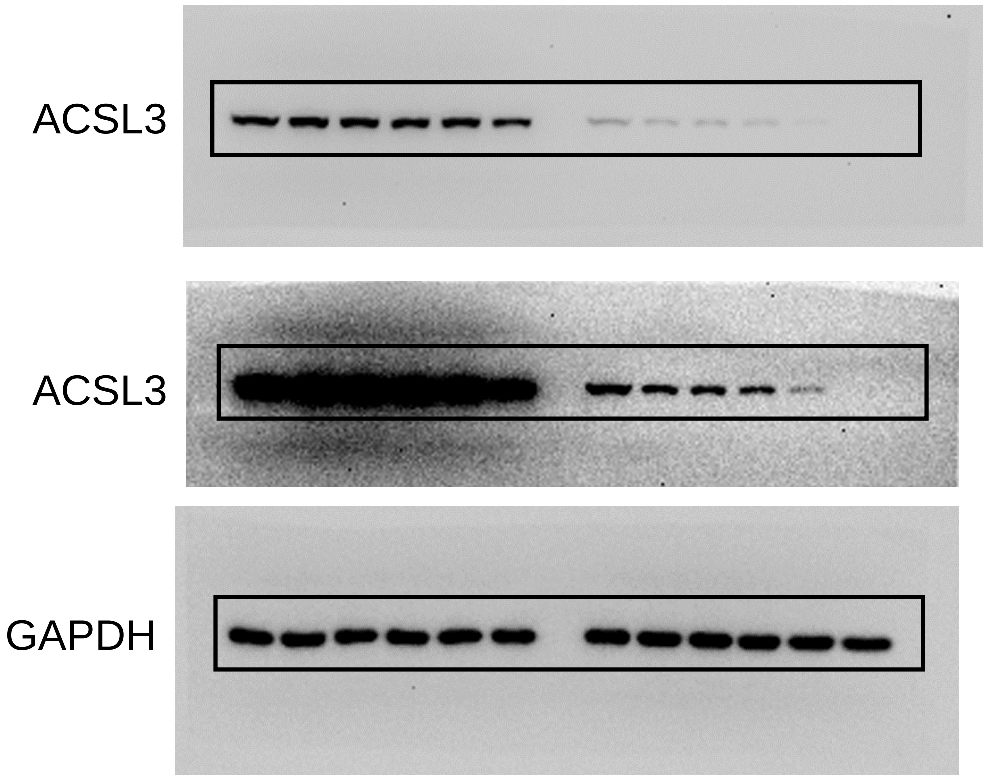

Figure 5H

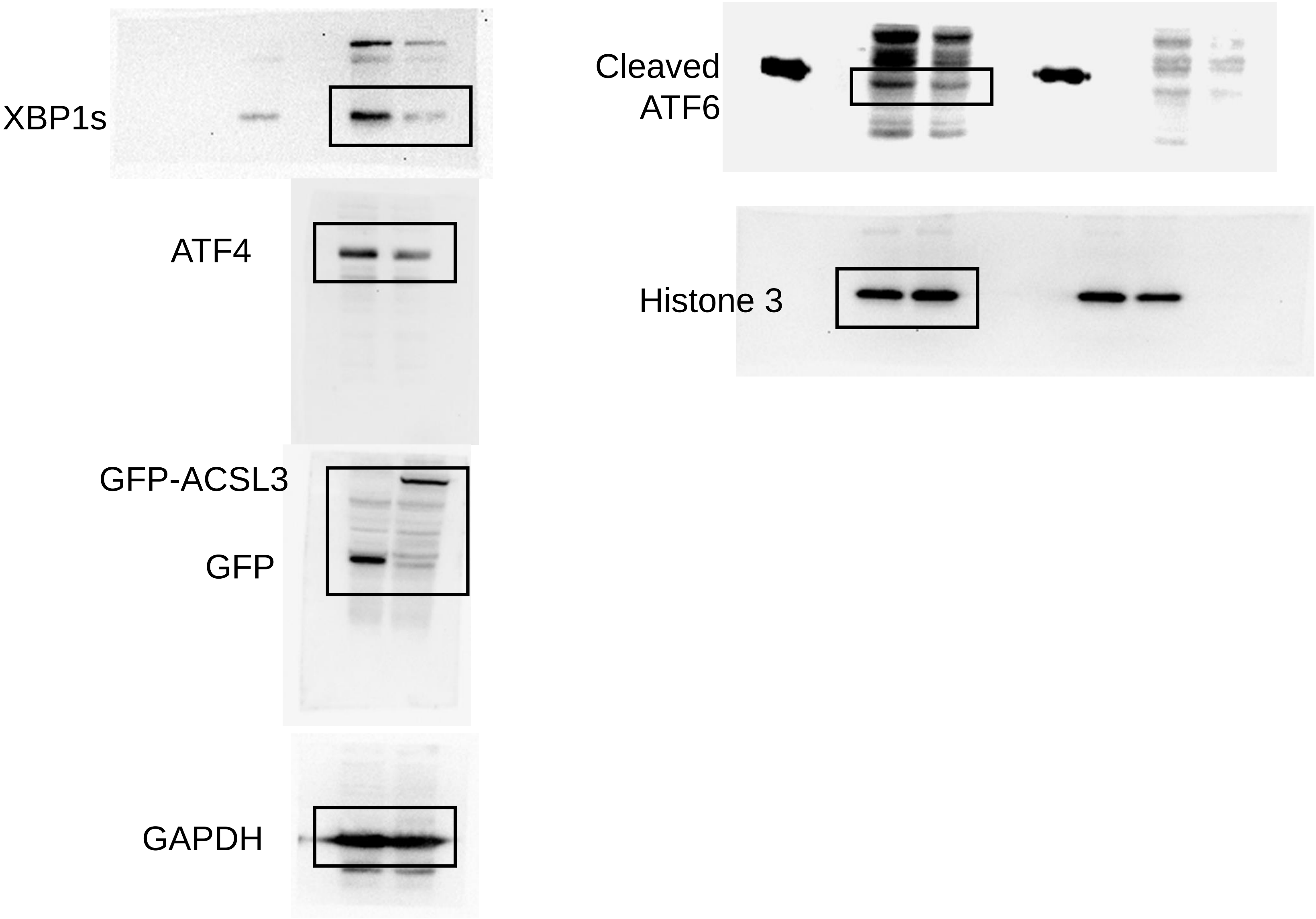

Figure 5J

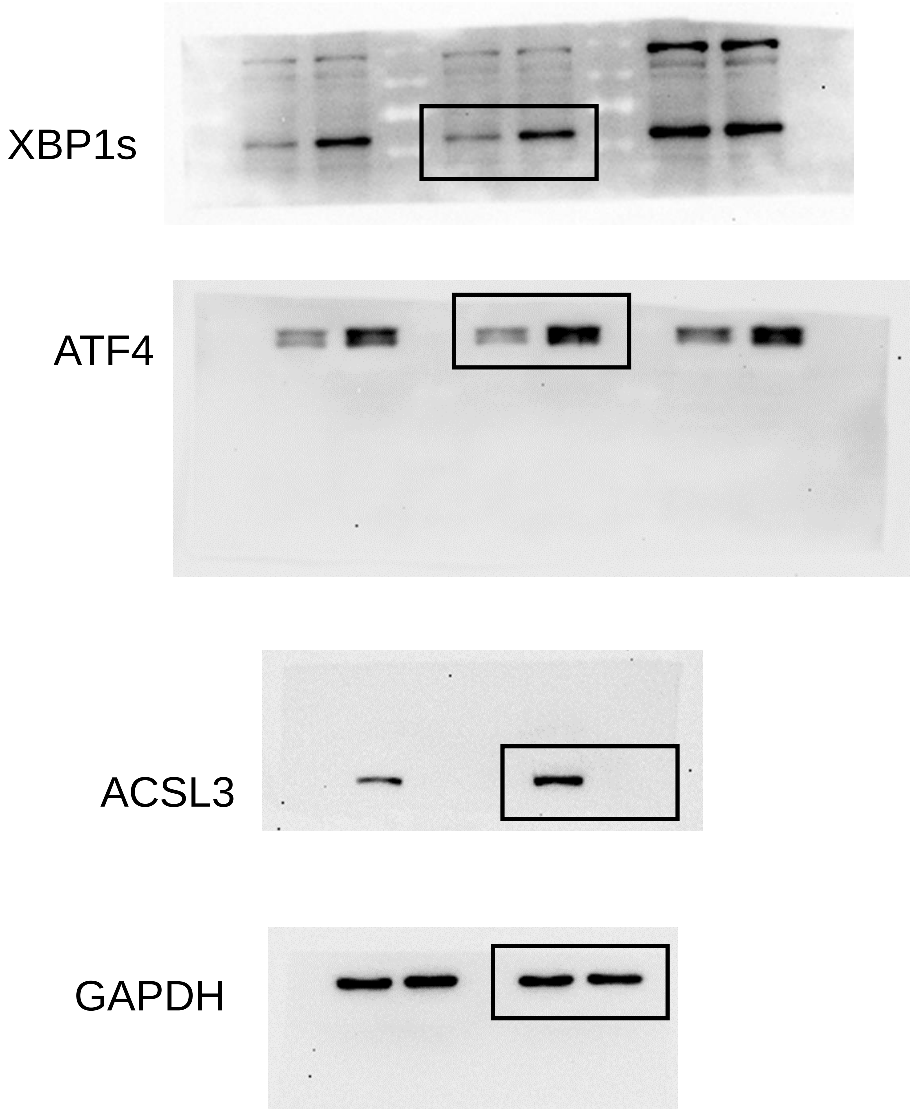

Figure 6H

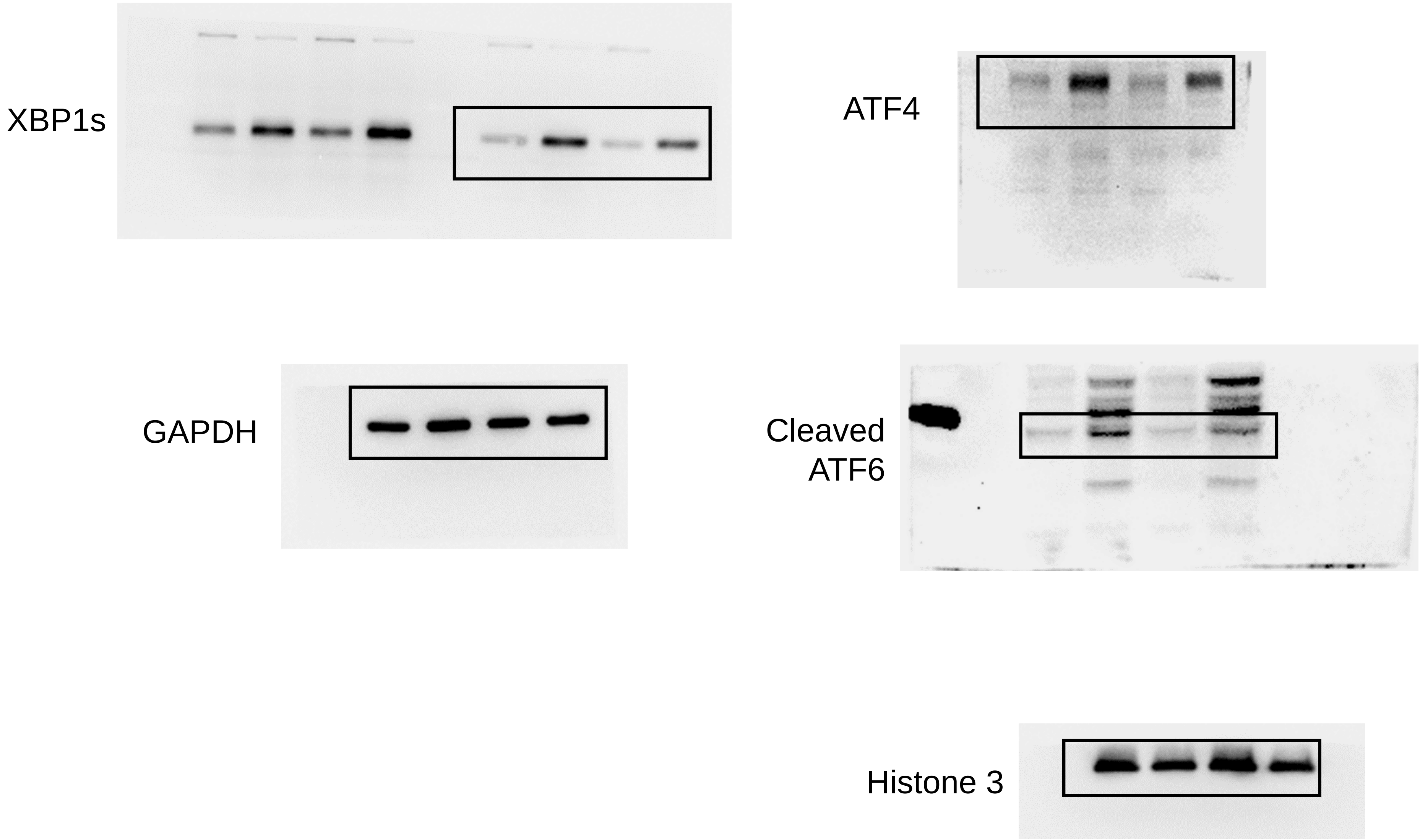

Figure 7F

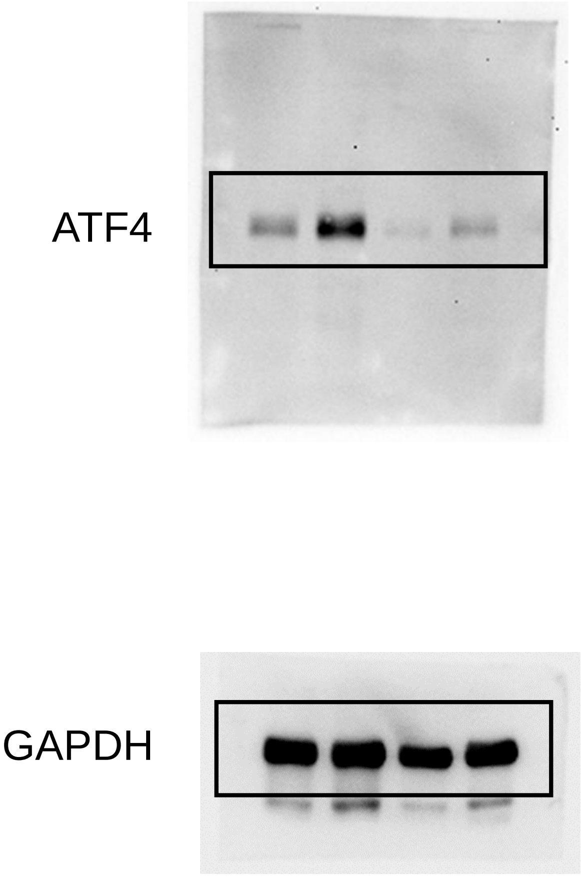

Figure 7G

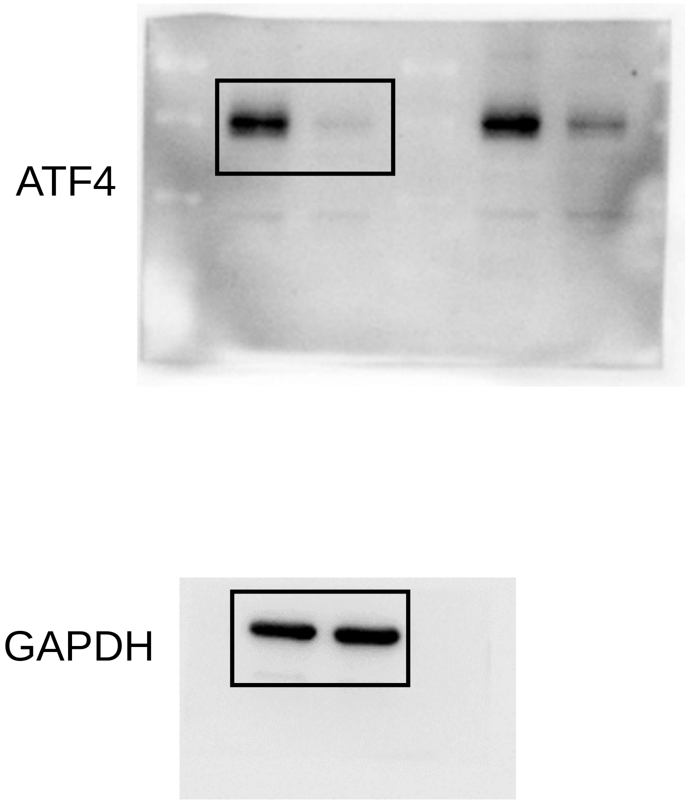

Figure S5C

XBP1s

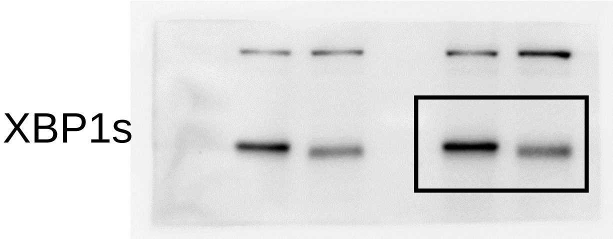

GAPDH

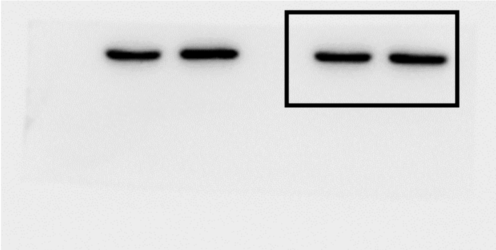

Figure S5D

ATF6

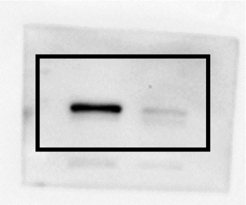

GAPDH

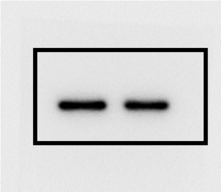

Supplement: Supplementary file 4 — Original Western Blot [file 41419_2025_8176_MOESM4_ESM.pdf]
